# Genome-wide analysis of the *Brachypodium distachyon* (L.) P. Beauv. *Hsp90* gene family reveals molecular evolution and expression profiling under drought and salt stresses

**DOI:** 10.1371/journal.pone.0189187

**Published:** 2017-12-07

**Authors:** Ming Zhang, Zhiwei Shen, Guoqing Meng, Yu Lu, Yilei Wang

**Affiliations:** College of Life Science, Heze University, Shandong, China; Hainan University, CHINA

## Abstract

The structure, evolution, and function of heat shock proteins 90 (Hsp90s) have been investigated in great detail in fungi and animals. However, studies on the *Hsp90* genes in plants are generally limited. *Brachypodium distachyo*n (L.) P. Beauv., as a model plant for cereal crops, has become a potential biofuel grass. During its long evolution, the *Hsp90* gene family in *Brachypodium* has developed some strategies to cope with adverse environments. How the *Hsp90* gene family in *Brachypodium* evolved in different plant lineages and what its role is in plant responses to drought and salt stresses remains to be elucidated. We used a set of different bioinformatics tools to identify 94 *Hsp90* genes from 10 species representing four plant lineages and classified into three subgroups. Eight *BdHsp90* genes were detected from *B*. *distachyo*n. The number of exon-intron structures differed in each subgroup, and the motif analysis revealed that these genes were relatively conservative in each group. The fragments duplication and tandem duplication, which are the prime powers for functional diversity, generally occurred during the duplication of the whole plant genome. Transcriptional analysis of the *BdHsp90* genes under salt and drought stress conditions indicated that the expression of these genes was delayed or increased at different stress time points; The expression was more affected in that of *Bradi3g39630*, *Bradi4g06370*, and *Bradi1g30130*. Our findings suggest the involvement of BdHsp90s in plant abiotic stress response, and further consolidate our views on the stress response mechanism of Hsp90 in general.

## Introduction

Plants are often exposed to various abiotic stresses, including drought, salinity, light intensity, heat shock, chilling, and chemical pollutants. These stresses act simultaneously on plants, causing cell injury and inducing many responses, such as osmotic and oxidative stress responses [1]. Various land plants have developed specific responses or tolerance mechanisms to cope with adverse environments. Among them, many stress proteins, such as heat shock protein (Hsp) chaperones, are induced to guard cells against these harmful stimuli [2].

The Hsps are classified into five categories based on their molecular weight, Hsp100 (e.g., 100 kDa), Hsp90, Hsp70, Hsp60, and small Hsp (sHsp). The 90-kDa heat shock protein (Hsp90) is an ATP-dependent molecular chaperone, with a highly conserved sequence from bacteria to higher eukaryotes and homologs in different organisms [3]. The core structure of Hsp90 contains the N-terminal ATP binding domain, the middle domain, and the C-terminal dimerization domain [3]. There is a notable feature of Hsp90s, which is a long, highly charged linker domain between the N-terminal and the middle domain in eukaryotic organisms [4].

Hsp90s are involved in regulating and maintaining the conformation of a variety of proteins, as well as in protecting normal cells from stress stimuli [5]. In fungi and in animals, Hsp90s play extensive roles in stress signal transduction, such as the folding of steroid hormone receptors, protein kinases, and transcription factors, as well as the activation of a substrate to initiate stress signal transduction [6–8]. Recent studies on Hsp90s in plants have mostly focused on evolutionary analysis and physiological functions [9–11]. A number of *Hsp90* genes have been identified in many plants, and strongly up-regulated by temperature, drought, salinity, and heavy metal stresses [12–14]. A recent proteomics and phosphoproteomics analysis in plants revealed that a number of Hsp90 proteins are both present under drought and salinity stress and are probably involved in signal transduction during the response to stress [15–17].

Although the *Hsp90* genes from plants have been known for more than a decade, our understandings of the stress response mechanisms of plant Hsp90s, their roles as molecular chaperones, and their molecular interactions with other clients and co-chaperones is limited [18]. The Hsp90 system in plants differs from that in animals in that it has an additional subcellular compartment, the plastid; Rapid molecular responses are other character subjected to sudden environmental changes [19]. It is necessary to better understand the proteins that are involved in signal transduction and other stress processes and how they activated, processed, and trafficked within plant cells [20]. The study, therefore, focused on the Hsp90 protein family in *Brachypodium distachyon* (L.) P.Beauv. to provide a comprehensive sequence-based understanding of the different family members in plants and to highlight the stress responses under drought and salt conditions. The similarities and differences between Hsp90 proteins of different plant origin were explored. The findings of this study provide a basis for functional analysis of Hsp90 in plants.

## Materials and methods

### Sequence retrieval and identification

*Hsp90* gene families were identified from 10 species representing four plant lineage from unicellular green algae to multicellular plants. The first search was performed using “Hsp90” as a keyword in the Phytozome v12.1 (https://phytozome.jgi.doe.gov/pz/portal.html) database. Seven *Arabidopsis thaliana Hsp90* genes were first searched and then used as a query in the BLAST against phytozome v12.1. E value of candidate sequences was below 1E-10, and redundant parts were excluded manually. Sequences were collected from the following four major plant lineages: the unicellular green algae *Chlamydomonas reinhardtii*; the moss *Physcomitrella patens*; the monocotyledonous angiosperms *B*. *distachyon*, *Oryza sativa* (rice), *Triticum aestivum* (wheat) and *Zea mays* (maize); and the dicotyledonous angiosperms *A*. *thaliana* (thale cress), *Glycine max* (soybean), *Medicago truncatula* (legume), and *Gossypium raimondii* (cotton). Candidate sequences were further confirmed by Pfam (http://pfam.xfam.org/) [20] and checked by SMART (http://smart.embl-heidelberg.de/) [21,22]. Finally, we obtained all protein and corresponding coding sequences (CDS) and genomic sequences of the *Hsp90* genes.

### Gene chromosomal location and phylogenetic analysis

Locations of 94 *Hsp90* genes were mapped by the MapInspect program and further modified manually. Phylogenetic trees were executed by Bayesian inference using the Markov Chain Monte Carlo (MCMC) method [23]. Initially, multiple sequence alignments were performed for full proteins based on the MUSCLE program (http://www.ebi.ac.uk/Tools/msa/muscle/) [24,25]. Bayesian inference phylogenyetic construction was performed by MrBayes v 3.2 using General Time Reversible (GTR) model with Γ distributed rates (gamma-distributed rate variation) [26, 27]. The set conditions of bayesian analysis are mcmc ngen = 8×10^5^ and samplefreq = 100. As the average standard deviation was below 0.01, the program was terminated. After discarding the burn-in samples, the remaining data were used to generate a Bayesian tree, which was shown by using FigTree v1.4.2.

### Exon-intron structure, conserved motif, chemical character analysis

The exon-intron structure of *Hsp90* genes was obtained by the online Gene Structure Display Server v2.0 (GSDS: http://gsds.cbi.pku.edu.cn) with CDS and genomic sequence [28]. The MEME program (Multiple Em for Motif Elicitation v4.10.2, http://meme-suite.org/tools/meme)[29,30]was used to identify conserved motifs in the candidate Hsp90 protein sequences. The parameters were as follows: number of repetitions = zero or one, maximum number of motifs = 10, and optimum motif width constrained = 6–50 residues. The Hsp90 pI/Mw was determined by the Compute pI/Mw tool (http://web.Expasy.org/compute_pi/) [31].

### Dating the duplication events

Tandem duplications were involved in multiple members of this family within the same or neighboring intergenic regions, and clustered these genes together with a maximum of 10 extra genes [32]. Segmental duplications of each *Hsp90* gene were queried in the Plant Genome Duplication Database (PGDD, http://chibba,agtec.uga.edu/duplication/). To calculate the occurrence of segmentally duplicated genes, the Ks value was searched in PGDD under the following conditions: Ks > 0, Ks ≤ 1, and anchor number was set to ≥3 between the same species. Based on synonymous substitutions per year (λ), the number of segmentally duplicated genes was 6.5 × 10^−9^ for *B*. *distachyon* [33], 6.5 × 10^−9^ for rice [34], 6.1 × 10^−9^ for soybean [33], 1.5 × 10^−8^ for *A*. *thaliana* [35], and 1.5 × 10^−8^ for cotton [36]. The approximate age (T) of duplication events of *Hsp90* gene pairs was then calculated using the equation T = Ks/2λ [37].

### Plant materials and stress treatments

The uniform seeds from the diploid inbred line of Bd21 were surface sterilized using 75% alcohol and 15% sodium hypochlorite, and then rinsed three times in sterile water. Subsequncely, the seeds were submerged in water (26°C) in complete darkness for 3 days. On the fourth day, seedlings were transferred to plastic pots containing full-strength Hoagland solution. The conditions were set at 16/8h (light/dark) photocycle, 28/26°C (day/night) and 70% relative humidity. Seedlings with three leaves were set in the following conditions: salinity stress (200 mM NaCl) and moderate drought stress [20% (w/v)]polyethylene glycol 6000 (PEG 6000). Leaf and root samples of control seedlings were harvested at 0h. The corresponding samples of treated seedlings were harvested at 12, 24, and 48h. Each sample was collected from 20 plants, with three replicates. All samples were immediately stored at -80°C until used.

### Total mRNA extraction and qRT-PCR analysis

Total RNA was isolated from frozen samples using TRIzol Reagent (Invitrogen) according to the manufacturer's instructions. Genomic DNA removal and cDNA synthesis were operated by using a PrimeScript®RT reagent Kit with gDNA Eraser (TaKaRa). Gene-specific primers of each *Hsp90* gene in *B*. *distachyon* were designed using the on-line tool Primer3Plus (www.bioinformatics.nl/cgi-bin/primer3plus/primer3plus.cgi) [38]. The primers were examined by blasting primer sequences in the NCBI database (http://www.ncbi.nlm.nih.gov/tools/primer-blast /index.cgi?LINK_LOC=BlastHome), and all primers were specifically in accordance with the respective sequence of its targeted gene. The primer sequences for the qRT-PCR assays are listed in S1 Table. Transcription levels of each *Hsp90* gene in *B*. *distachyon* were quantified by a CFX96 Real-Time PCR Detection System (Bio-Rad) using the intercalating dye SYBR-green and following the 2(-Delta Delta C(T)) method [39]. The *B*. *distachyon* constitutively expressed Ubiquitin gene was used as a reference for normalization [40]. qRT-PCR was performed in a 20 μL volume reaction system containing 10μL 2× SYBR®Premix ExTaq™ (TaKaRa), 2μL 10-fold diluted cDNA, 0.15μL of each gene-specific primer and 7.7μL ddH_2_O. The PCR conditions were as follows: 95°C for 3 min, 40 cycles at 95°C for 20s, 61°C for 15s and 72°C for 10s. Triplicates for each PCR and three biological replicates were performed for each gene. The results for the qRT-PCR assays are listed in S1 Table. The qRT-PCR efficiency was determined by five serial ten-fold dilutions of cDNA. Statistical analyses were conducted using independent Student’s t tests with SPSS statistics software (version 17.0). The hierarchical clustering results were performed using Cluster 3.0 and TreeView softwares.

## Results

### Identification of *Hsp90* genes in *B*. *distachyon* and in the other nine representative plant species

To clarify the origin and evolution of the *Hsp90* genes in plants, we identified 10 species representing four major plant lineages. A total of 94 *Hsp90* genes were obtained (S2 Table). Each gene contained conserved HATPase_c and HSP90 domain (S3 Table), and PF02518 and PF00183 families by Pfam (S3 Table). Among all *Hsp90* genes, three were identified in the unicellular green algae, 11 in the moss, 42 in the monocotyledonous angiosperms and 38 in the dicotyledonous angiosperms (S1 Fig). Furthermore, the *Hsp90* genes identified in the 10 plant species encode proteins ranging from 278 to 1054 amino acids (aa) in length, but the majority are approximately 695 aa. The predicted isoelectric points range between 4.79 and 6.28, and Molecular weight range between 40.6 and 123.6 kDa. The detailed information of these identified *Hsp90* genes were listed in S2 Table.

### Phylogenetic relationships of the *Hsp90* gene family

**Fig 1:**
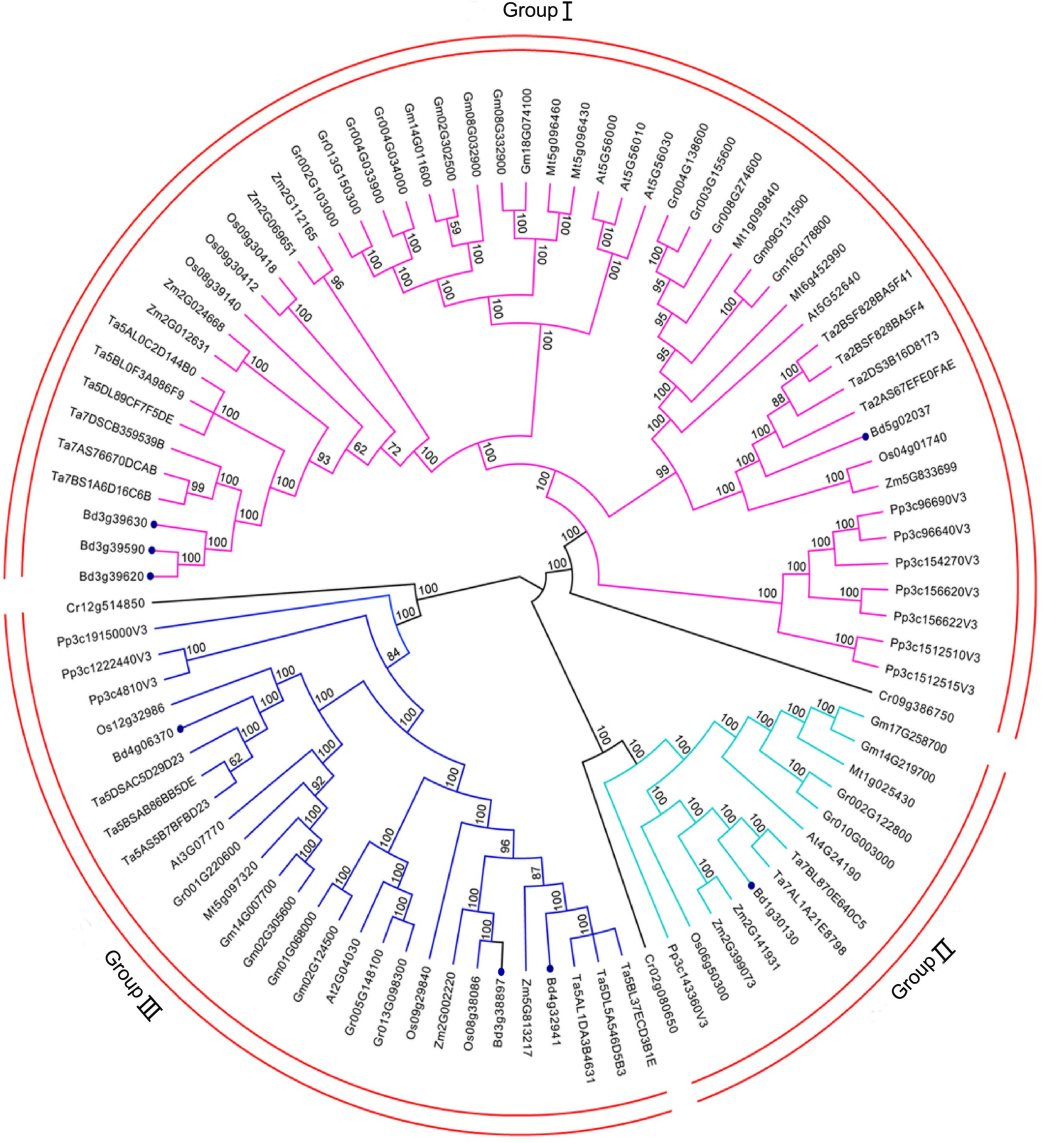
Phylogenetic relationships of Hsp90 gene family. A total of 94 protein sequences of Hsp90 gene family identified from ten species vary from unicellular green algae to multicellular plants were aligned with MUSCLE program, and the phylogenetic tree was constructed based on Bayesian inference using Markov Chain Monte Carlo (MCMC) methods. The red arcs indicate different subgroups of Hsp90 genes. Hsp90 genes of B. distachyon are indicated by filled purple dots.

The Bayesian inference, a standard approach for the estimation of branch support, was executed by MrBayes as posterior probabilities within the time run [41]. To deeply explore the evolutionary relationships of *Hsp90* genes within various plant species, the full amino acid sequences of 94 identified proteins were to execute multiple sequence alignment using the MUSCLE program. An unrooted phylogenetic tree was constructed using the Markov Chain Monte Carlo (MCMC) method based on Bayesian inference (Fig 1). Finally, the resulting tree topology classified these plant genes into three subgroups. Interestingly, the genes in *C*. *reinhardtii* were lined the first in each group. Moreover, the distribution of *Hsp90* genes from different individual species had slight differences and unique patterns in each group (Fig 1). Of the 94 plant *Hsp90* genes, 53 is in Group I, 14 in Group II and 27 in Group III. From an evolutionary perspective, the resulting phylogram (S2 Fig) is in agreement with the timeline of plant evolution: unicellular green alga occurred first and was followed by the moss, monocotyledonous angiosperms, and dicotyledonous angiosperms. Within *A*. *thaliana*, four genes were in Group I, one in Group II, and three in Group III. Almost the same distribution pattern was observed for the *Hsp90* gene family members from *A*. *thaliana* (Fig 1). Overall, the distribution characters of the *Hsp90* gene family were similar among the 10 plant species.

### The analysis of structural characters of the *Hsp90* gene family

**Fig 2:**
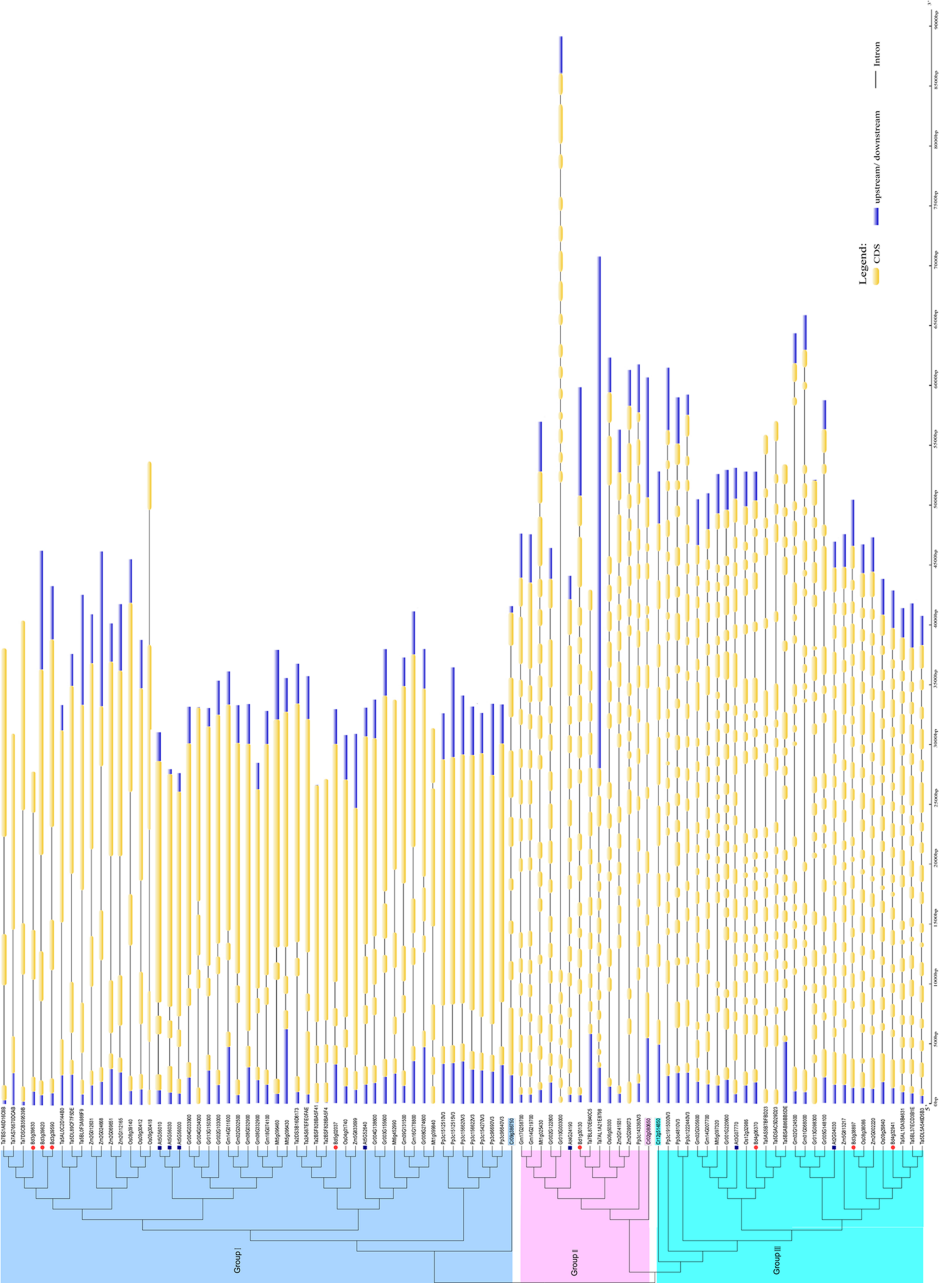
Introns and the corresponding exon sequences within individual Hsp90 gene paralog listed in Neighbor-joining phylogenetic tree. Hsp90 genes of B. distachyon are indicated by filled purple dots. Hsp90 genes of A. thaliana are indicated by filled purple dots. Colorbar indicates the number of introns contained in Hsp90 genes.

To investigate the possible molecular mechanisms underlying the expansion of the *Hsp90* gene family, the exon-intron structure in all chosen plant lineages was investigated using the online GSDS. The relative length of introns and the corresponding exon sequences within individual *Hsp90* gene paralogs are listed on the neighbor-joining phylogenetic tree (Fig 2). Our results revealed that in the same group, the gene members had similar exon-intron structures. Although the number of introns in individual *Hsp90* genes ranged from 1 to 21, there were no significant differences in the number of introns between individual genes within the same group, except for those in *C*. *reinhardtii* (Fig 2). Group I, the largest family comprising 56% of the genes in the subfamily, had 1–3 introns. Group II, the smallest family, which contained 16% of the genes in the subfamily, had 14–16 introns. The number of introns in Group III, which included 28% of the genes, ranged between 17 and 21. In contrast, the number of introns in genes of the unicellular green alga (Cr) did not follow these patterns, and were 7, 11, and 8 introns in Group I, Group II, and Group III, respectively (Fig 2).

**Fig 3:**
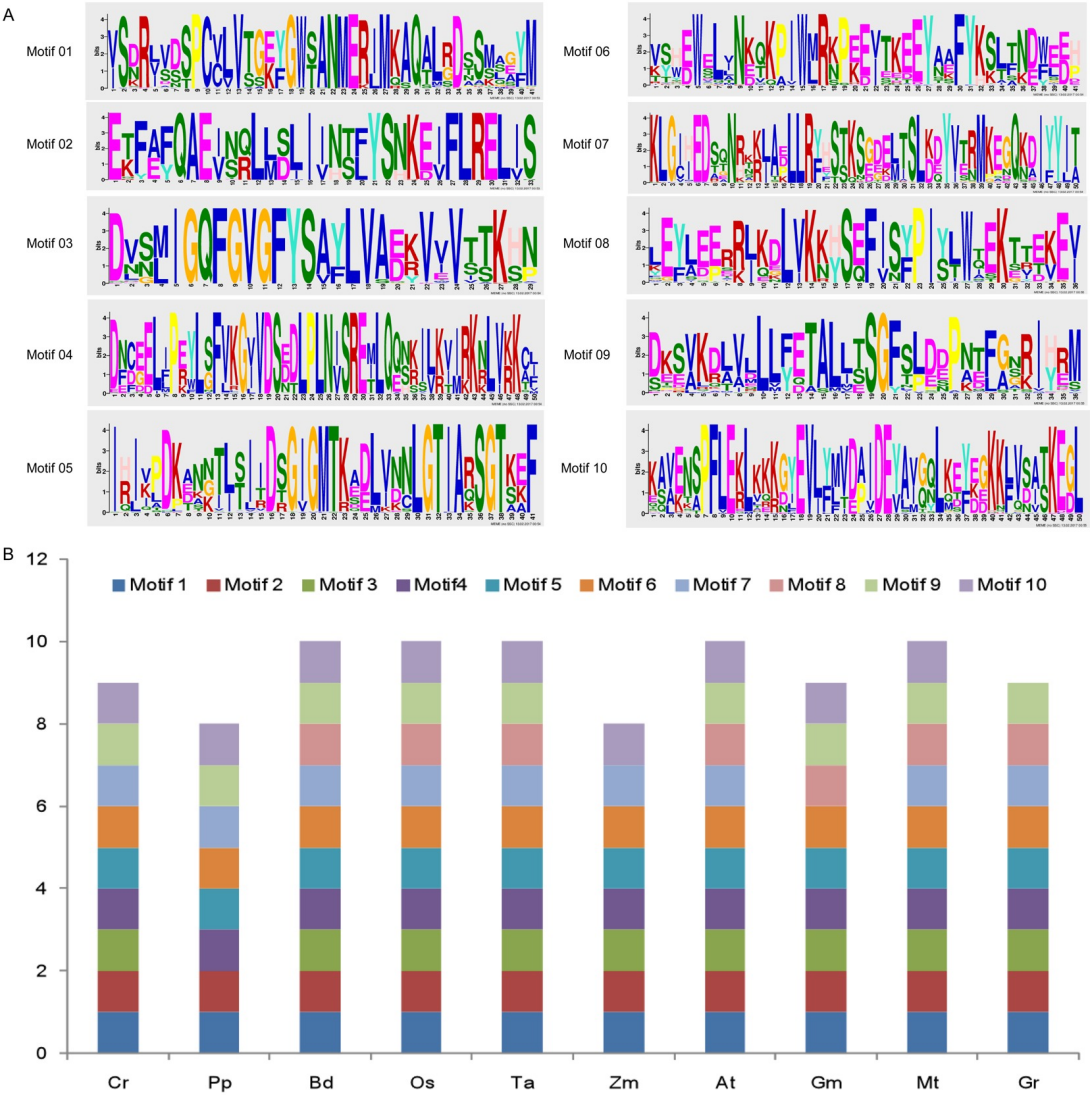
The schematic diagram of Hsp90 protein motifs of all Hsp90 genes and each species from MEME.

To explore the possible function for the structural evolution of the *Hsp90* gene family, the conserved protein motifs were identified in these genes using MEME. Ten conserved motifs were detected in the 94 *Hsp90* genes (Fig 3).The structure of individual *Hsp90* gene paralogs is shown in Fig 3. The number of motifs contained in the individual *Hsp90* gene was primarily 7–10. Similarly with the patterns of introns observed in each gene, the number of conserved motifs was relatively consistent within individual *Hsp90* gene paralogs. Up to 50% of the *Hsp90* genes contained 10 motifs, whereas the remaining genes contained 8 motifs (25% of the genes) and 9 motifs (25% of the genes).

### Chromosomal distribution and gene duplication events of the *Hsp90* gene family

**Fig 4:**
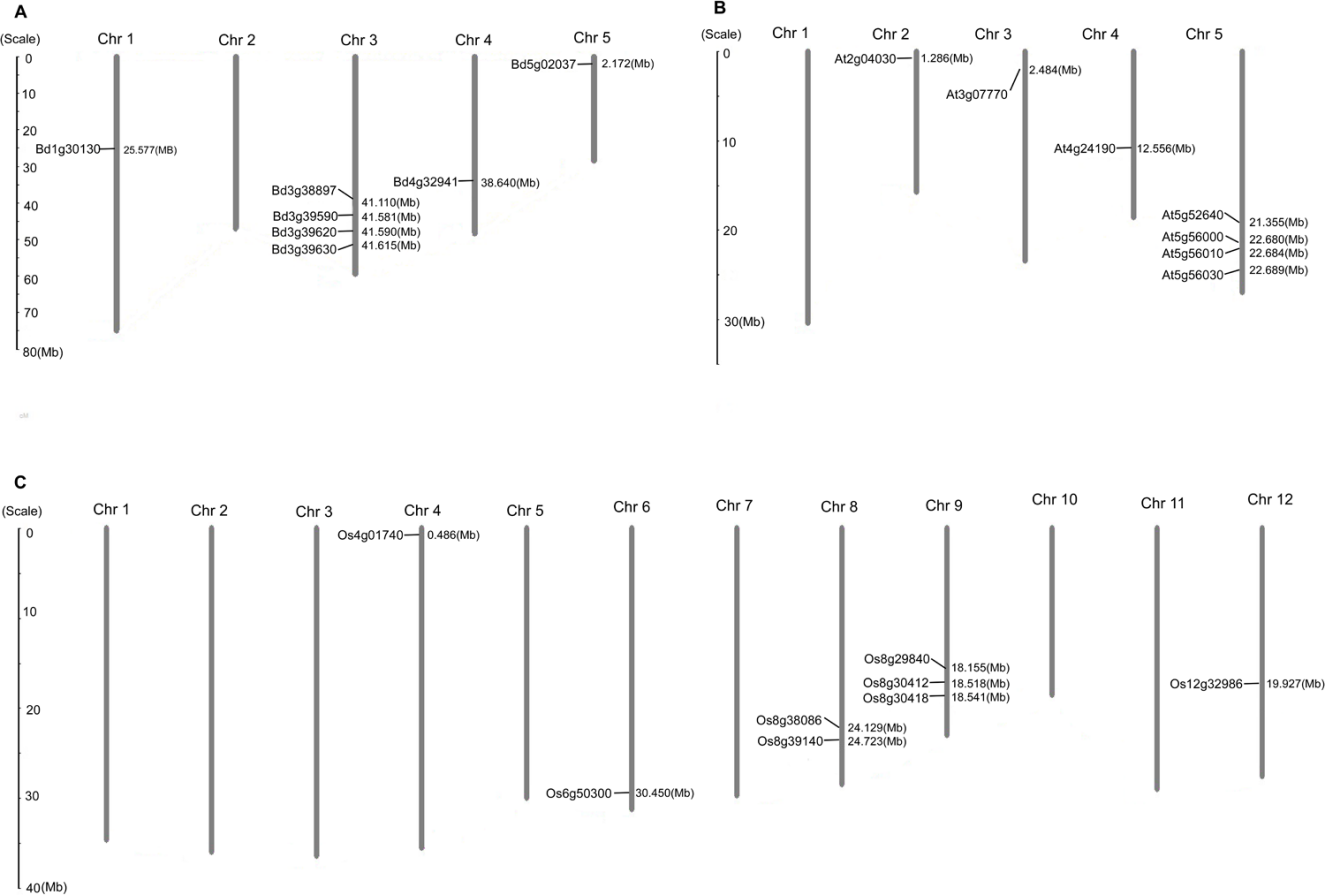
Chromosome distribution of Hsp90 genes in B. distachyon, A. thaliana and O. sativa. (A). The chromosome distribution of Hsp90 genes in B. distachyon. (B). The chromosome distribution of Hsp90 genes in A. thaliana. (C) The chromosome distribution of Hsp90 genes in Oryza sativa. The chromosome numbers are indicated at the top of each bar and size of a chromosome is indicated by its relative length.

The locations of *Hsp90* genes in *B*. *distachyon*, *A*. *thaliana*, and *Oryza sativa* were determined using MapInspect, and the results are shown in Fig 4. The eight *Hsp90* genes from *B*. *distachyon* were mapped on four chromosomes (chromosome 01, 03, 04, and 05), four of eight genes were mapped on chromosome 03. Similarly, the *Hsp90* genes of *A*. *thaliana* and *O*. *sativa* were not distributed in the whole genome (Fig 4). However, the gene numbers on each chromosome were directly proportional to the length of the corresponding chromosome, suggesting that the *Hsp90* genes in multiple plant species have no obvious chromosomal preferences. Furthermore, the genes were clustered in certain chromosomal regions or dispersed individually in other locations, thus corroborating the mapping results reported in other plants [42, 43].

There are the three dominant evolutionary events in gene duplication, including segmental duplication, tandem duplication, and transposition (retroposition and replicative transposition) [44], among which segmental duplication and tandem duplication commonly occur in plants based on polyploidy and unequal crossing-over, respectively[40]. To gain some insights into the expansion pattern of the *Hsp90* genes, we identified tandem duplicates and segmental duplication clusters based on the gene locus and searched the PGDD [45, 46] to locate duplicated pairs. The results showed that 13 out of the 94 genes (13.8%) in this family were tandem repeats, indicating that tandem duplications have contributed to the expansion of the *Hsp90* gene family. A total of 22 genes (23.4%) showed segmental repeats (S4 Table), suggesting that segmental duplication events played an important role in the evolution of the *Hsp90* superfamily in those species. The details were as follows: one pair of genes were identified as segmental duplications (*Bd3g38897* and *Bd4g32941*) in *B*. *distachyon*, two pairs of segmentally duplicated genes were identified in rice (*Os08g38086* and *Os09g29840*, *Os08g39140* and *Os09g30412*), and nine pairs of segmental duplications were found in soybean. The precise information of gene duplication events in these species are listed in Table 1.

**Table 1:** Segmental duplication events of some plant species.

Gene pairs		Anchor Numbers	Ks	Time	GWD (mya)	References
			(means ± s.d.)	(mya)		
Bd3g38897	Bd4g32941	5	0.804 ± 0.149	61.8	50–70	Kellogg *et al*., 2001[52]; Gaut *et al*., 2002[53]
Os08g39140	Os09g30412	3	0.683 ± 0.200	57.1	53–94	Yu *et al*., 2005[34]
Os08g38086	Os09g29840	4	0.850 ± 0.162	65.4		
Gm02G302500	Gm08G332900	7	0.611±0.186	50.8	5–13,59	Schmutz *et al*., 2010[54]
Gm08G332900	Gm14G011600	7	0.584 ± 0.146	47.9		
Gm08G332900	Gm18G074100	13	0.128 ± 0.039	10.5		
Gm02G302500	Gm14G011600	24	0.117 ± 0.043	9.6		
Gm14G011600	Gm18G074100	6	0.608 ± 0.190	49.8		
Gm02G302500	Gm18G074100	6	0.613 ± 0.193	50.8		
Gm14G219700	Gm17G258700	15	0.158 ± 0.139	12.9		
Gm01G068000	Gm02G124500	4	0.220 ± 0.167	18.2		
Gm02G305600	Gm14G007700	19	0.098 ± 0.025	8.1		
Gr003G155600	Gr004G138600	3	0.660 ± 0.147	19.6	13–20	Wang *et al*., 2012[55]
Gr002G103000	Gr013G150300	3	0.512 ± 0.230	17.1		
Gr002G122800	Gr010G003000	3	0.605 ± 0.168	20.1		

MYA: million years ago

### Subcellular localization and evolution relationships of the *Hsp90* gene family

**Fig 5:**
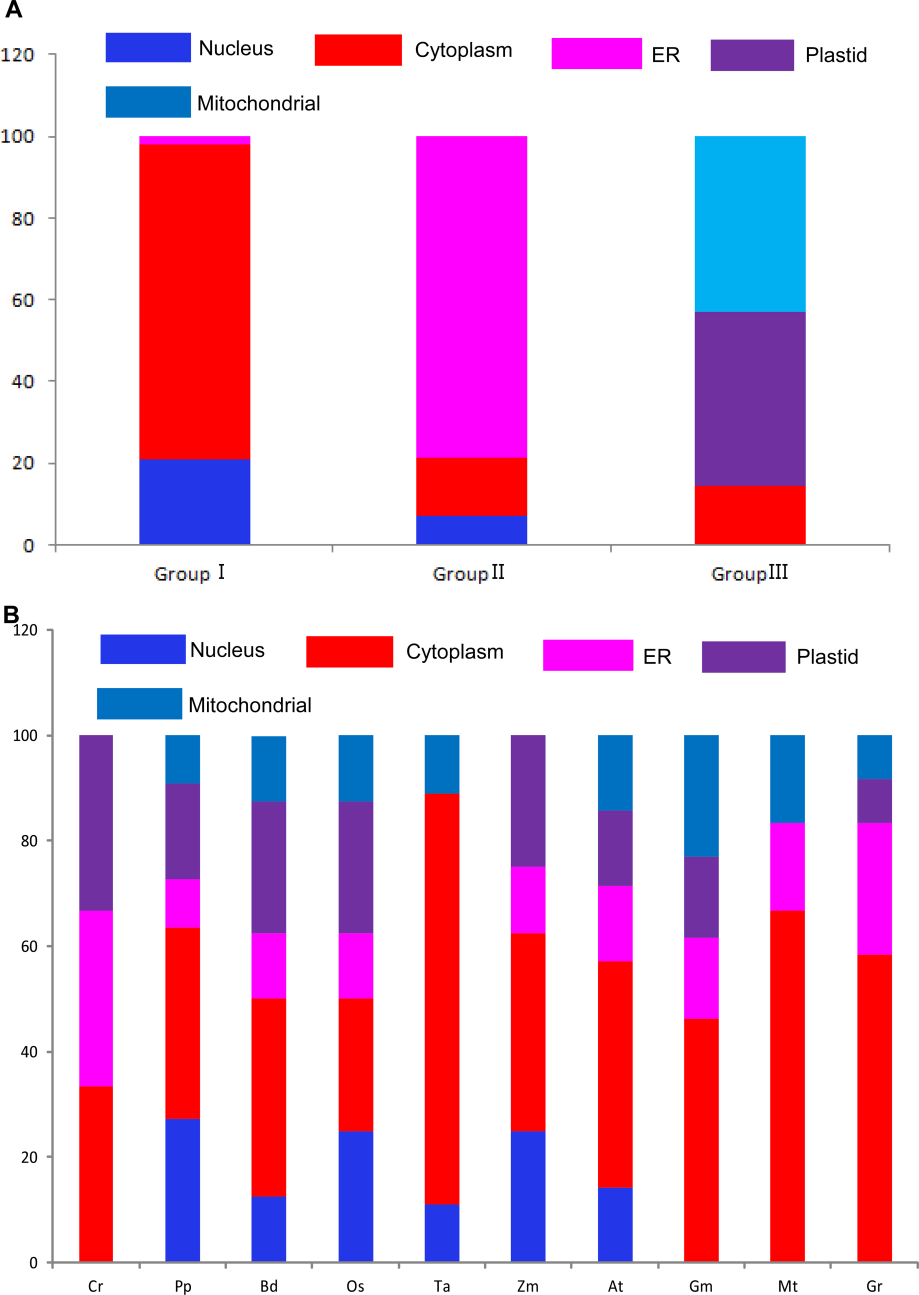
Subcellular localization of each subgroup and species by TargetP1.1, WoLF PSORT and Predotar v. 1.03 on-line tools.

Subcellular localization is a key characteristic of protein functional research. It can take part in the cell activity and function efficiently in correct subcellular location [47]. In our study, based on three bioinformatics tools, including TargetP1.1 [48], WoLF PSORT and Predotar v. 1.03[49], subcellular localization was conducted in 94 Hsp90 proteins (Fig 5 and S5 Table). Interestingly, based on the above analysis, the results showed that these Hsp90s in Group I are largely distributed in the nuleus (20%) and cytoplasm (80%). Moreover, all of their ends contained the conserved MEEVD sequence. The Hsp90s in Group II were mostly districted in the ER (78%), and all of their ends contained KDEL sequence, which is a specific retention sequence in the endoplasmic reticulum [49]. The Hsp90s in GroupIII were mostly districted to the chloroplasts (40%) and mitochondria (45%), and all of their ends contained extra sequences differed from Group I and Group II. The results were in accordance with previous results [19].

### Expression profiles of *Hsp90* genes in root and leaf tissues under osmotic stress

**Fig 6:**
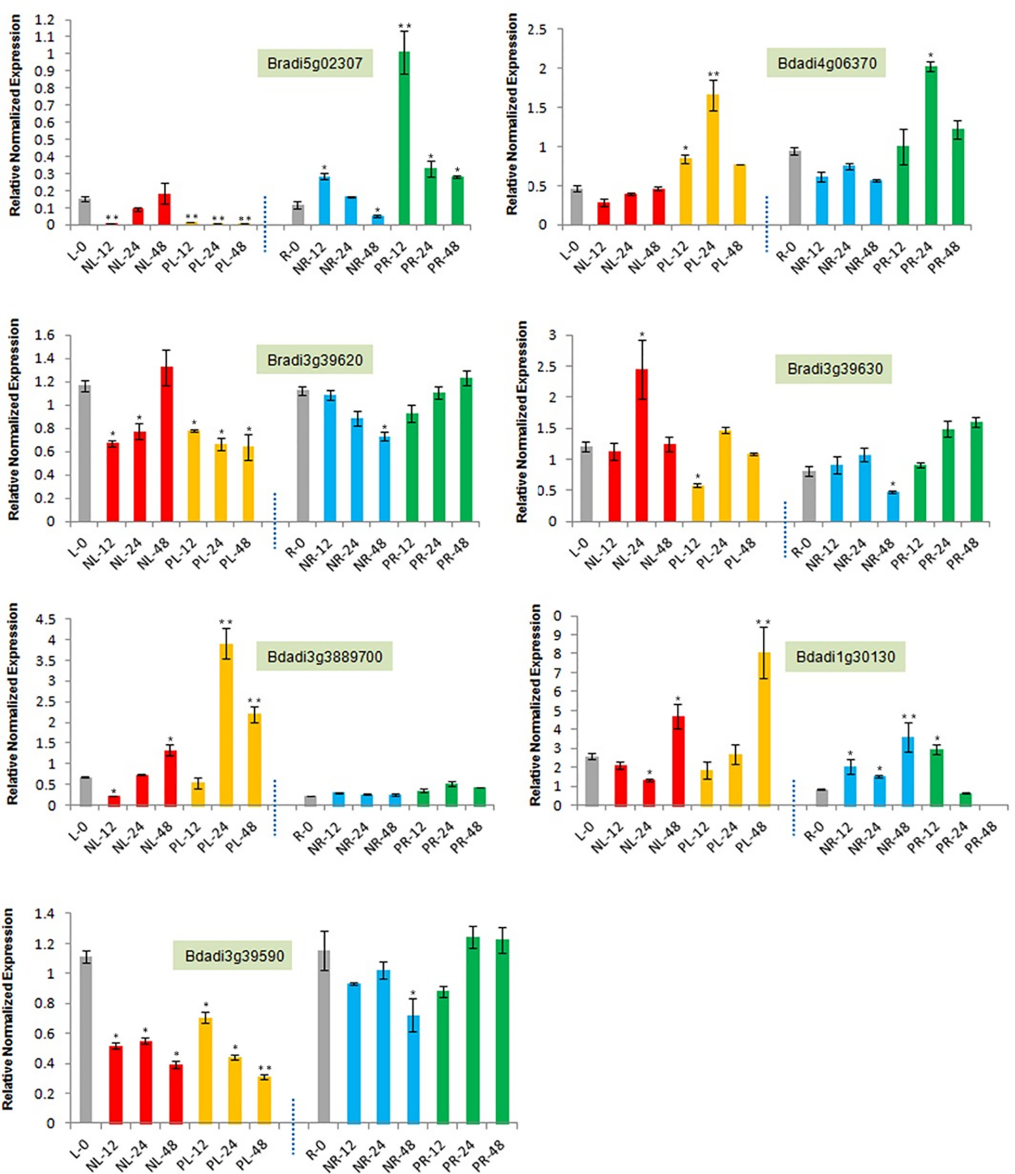
The relative expression profiling of seven B. distachyon Hsp90 genes in root and leaf under drought and salt stresses (12h, 24h, 48h). L0: Leaf under untreated conditions. R0: Root under untreated conditions. NL: NaCl stress Leaf. PL: PEG6000 stress Leaf. NR: NaCl stress Root. PR: PEG6000 stress Root. Note: “*”for significant difference (P<0.05); “**”for highly significant difference (P<0.01).

**Fig 7:**
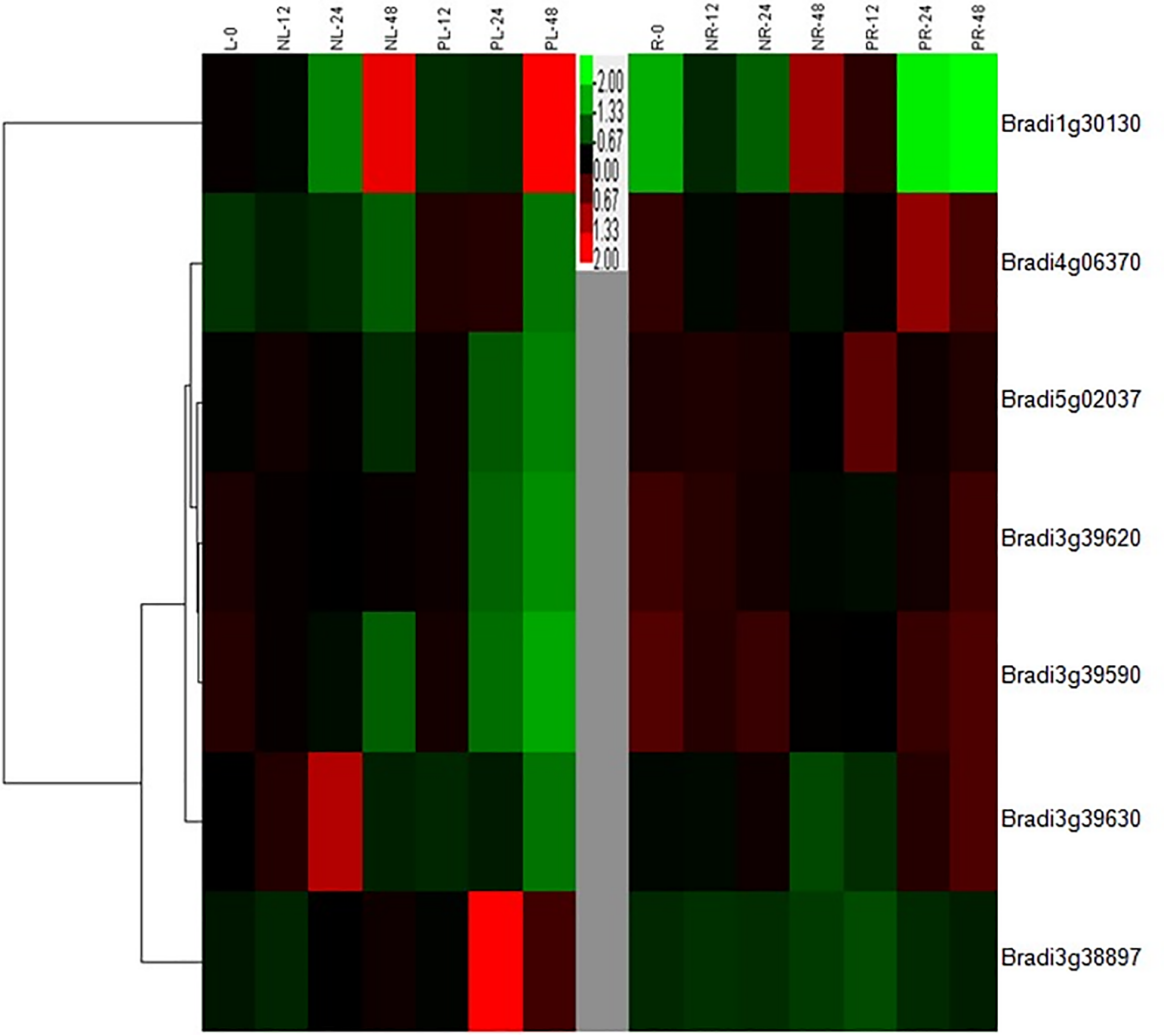
The hierarchical cluster (HCA) analysis of seven B. distachyon Hsp90 genes in root and leaf tissues under drought and salt stresses (12h, 24h, 48h). Colorbar indicates the amounts of Hsp90 gene expression.

We tested the response mechanism in two different stress conditions by firstly constructing eight *B*. *distachyon* genes primers using the on-line tool Primer3Plus [37]. Unfortunately, seven of eight genes were constructed of their primers and standard curve (S1 Table, S3 Fig). The expression profiles of the seven *Hsp90* genes in the root and leaf tissues of *B*. *distachyon* were analyzed using real time PCR (Fig 6, Fig 7). Under normal conditions, the expression level of *Hsp90* genes in the root is no higher than that in the leaf, except for *Bradi1g30130* (3.5-fold) and *Bradi3g3889700* (3-fold) (Fig 6). In addition, it is worth noting that the expression level of *Bradi3g3889700* in the root is lower than the expression level of the other six genes, while the expression level of *Bradi1g30130* was higher than that of the other six genes (Fig 6). These results indicate that the transcriptional profiles of the *Hsp90* genes in the *B*. *distachyon* have specific tissue expression.

Drought and salt stresses were two common types of abiotic stresses. The common response that they induced was that of osmotic stress, the same as heat stress. In our study, the expression pattern of four *Hsp90* genes in the leaf tissue showed first down-regulation and then up-regulation under salt stresses, when compared with the control condition, while *Bradi1g39590*, *Bradi5g02307* and *Bradi3g39620*generally showed down-regulation in most stress time points (Fig 6). Moreover, under drought stress, the expression pattern of three *Hsp90* genes, including *Bradi5g02307*, *Bradi3g39620*, and *Bradi3g39590* totally showed significant down-regulation, while the other four genes showed significant up-regulation in comparison to the control condition, especially in 24h or 48h points (Fig 6).

In root tissue, compared with control condition, *Bradi3g3889700* showed almost no expression changes, and *Bradi1g30130* showed significant up-regulation under salt stress, while the other five *Hsp90* genes showed a down-regulation pattern (Fig 6). Furthermore, under drought stress, *Bradi3g39620*, *Bradi3g39630* and *Bradi3g3889700* showed no expression changes, compared with the control. The other four genes showed obvious up-regulation pattern, especially in *Bradi5g02307*, *Bradi1g30130* and *Bradi4g06370*, which might be involved in stress responses. From the clustering analysis, and in contrast to the express pattern of other genes, *Bradi1g30130* showed significant up-regulation pattern under two stresses, especially in the 48h stress point (Fig 7), suggesting that it might participate in stress tolerance. *Bradi3g3889700*, as the leaf-specific stress response gene, showed no change in root tissue under the two stresses, which is consistent with its subcellular localization (chloroplast).

## Discussion

The *Hsp90* genes family, is large and has been identified in almost studied eukaryotic species. To date, the necessary roles of the *Hsp90* genes in higher plants have been investigated in response to biotic and abiotic stresses [10, 50]. In the current study, we explored the phylogenetic relationships and characters of the 94 *Hsp90* genes from 10 species using a variety of bioinformatics tools. To study the drought response profiles of the plant *Hsp90* genes, the eight *Hsp90* genes in *B*. *distachyon* were all checked for the expression analysis under drought and salt stresses using the real-time PCR method combined with the multiple sequence alignment. The results showed that the evolution of plant *Hsp90* genes is relatively conserved from unicellular green algae to dicotyledonous, while the expression profile of the *Hsp90* genes in different tissues of *B*.*distachyon* is divergent in response to drought and salt stresses.

### Evolution profile and duplication events of the *Hsp90* gene family

In this study, 94 *Hsp90* genes were selected from 10 species representing the four major plant lineages. From these results, we can see that *Hsp90* gene members in 10 plants species are different, while the number of the *Hsp90* genes in higher plants is more than that of lower plants. Using MCMC method based on Bayesian inference, phylogenetic trees were supported by high bootstrap values, and showed that these *Hsp90* genes belong to three major subgroups. These findings are in accordance with those of previous studies [9, 11]. Interestingly, three Cr occurred first in each group, suggesting that the characters of the *Hsp90* gene family were conserved throughout the evolutionary history. The development of the intron was an important process in genomic evolution, and an adaptive measure for speciation evolution [51]. Interestingly, our results showed that genes in each subgroup possess similar exon-intron structures with similar numbers of introns, while the genes in different subgroups manifested slightly different exon-intron structures. In addition, the pattern also can be found in the individual species. Using subcellular location analysis, *Hsp90* genes were almost all distributed in nuleus/cytoplasm in Group I. For Group II, the *Hsp90* genes were almost all located in the ER. Compared with above two groups, *Hsp90* genes in Group III were almost all located in the chloroplasts/mitochondria. Based on the exon-intron structure and subcellular location, we speculate that our evolution classification in plant Hsp90s is creditable.

Gene duplication events play critical roles in the gene evolution, and have developed into a major mechanism for the establishment of new functions [51]. In this study, 23.4% (22 of 94) *Hsp90* genes were considered to be derived from segmental duplication events, and these segmental duplication gene pairs occurred in the same subfamilies, suggesting that they can show new evolutionary functions after duplication. The estimated time of segmental duplication events ranged from 8.1 to 61.8 Mya, demonstrating that these *Hsp90* duplicated genes may experience the split in whole genome duplication for each species. For *B*. *distachyon*, there was only one pair segmental genes found (*BdGF14e/BdGF14b*), and it was estimated to be originated at about 61.8 Mya. As Table 1 was seen [52–55], the divergence time of Poaceae was approximately 50–70 Mya. The whole genome duplication of cotton, soybean and rice was approximately 13–20, 5–13(59) and 53–94Mya, respectively, suggesting that the duplication of *B*. *distachyon Hsp90* gene family occurred before the divergence of the genome from cotton, and approached to point of genome its divergence from rice and soybean.

### The expression profiling and possible response mechanism of the *B*.*distachyon Hsp90* genes in response to osmotic stresses

It is clear that the plant *Hsp90* gene family has divergent functions, and that they participate in a wide range of biological processes, especially occurring in response to abiotic stresses [56]. In addition, previous investigations suggest that plant *Hsp90* genes have different response mechanisms in response to different stresses. Most of the *Hsp90* genes perform their functions through participating in biological signaling pathways, such as the abscisic acid (ABA) signaling pathway and the ER stress signaling pathway [56, 57].

### The *B*.*distachyon* cytoplasm *Hsp90* genes in response to osmotic stresses through the ABA signaling pathway

The hormone ABA is a very important phytohormone that play important roles not only in seed germination and development, but also in response to a wide range of abiotic stresses, such as exogenous ABA, salinity, drought, temperature stresses [58]. The response mechanism of plants against many abiotic environmental stresses is through the ABA-dependent signaling pathway. [59–61].

Based on previous experiment results, Jacob P. *et al*, (2017)[62] provides a possible stress response mechanism of Hsp90 mediated with ABA signaling. In normal conditions, HsfA1s are bound by Hsp90/70 and their co-chaperones ABI5 (ABA insensitive five) and DREB2A (dehydration responsive element binding protein 2A) were linked through the E3 ligase DRIP1/2, and degraded by 26 proteasomes [63, 64]. Upon stress application, the high number of misfolded and denatured proteins triggers the recruitment of Hsp90/70 to its client and releases the HSFA1s [62]. Especially, heat and drought stresses will lead to phosphorylated E3 ligase degradation due to SnRK2 activation, and DREB2A and ABI5 accumulation [64–66], which can then enter the nucleus, cooperatively or separately bind their target DNA, and activate the expression of the target genes [62]. In our study, based on cellular location and evolution classification, four *Hsp90* genes in the *B*. *distachyon*, including *Bradi3g39590*, *Bradi3g39620*, *Bradi3g39630*, and *Bradi5g02307*, were distributed in the cytoplasm (Fig 1, Fig 4). Of the four genes, the expression pattern of *Bradi3g39620* and *Bradi5g02307* generally showed an up-regulated trend in response to osmotic stress, while the other two genes showed down-regulated trends, which might be involved in plant development under stress conditions. Moreover, the evolution distribution of *Bradi3g39620* was similar with that of *At5g56100* (AtHsp90.2) and relatively homologous (72%), and the evolution distribution of *Bradi5g02307* was similar to that of *At5g52640* (AtHsp90.1), suggesting that the up-regulation of the two cytoplasm *Hsp90* genes may participate in the osmotic stress response of ABA signaling through freeing of the relative transcript factors, which activate downstream gene expression in stress response and tolerance. Moreover, *OsHSP90-2* and *OsHSP90-4* were also found up-regulated to be drought, cold, heat and salt stresses [67], which is consistent with our results. However, in a report that constitutively reduced cytosolic Hsp90 by using siRNA method, the expression of genes generally responsible for stress responses such as ABA stimulus, drought stress, and jasmonic acid response were enhanced [68]. These results showed that some cytoplasm *Hsp90* genes might be the stress response “monitor”, by positively and passively fining and regulating to take part in cell protection and control against adverse environments.

### The ER *B*.*distachyon Hsp90* genes response to osmotic stresses through the ubiquitin proteasome system (UPS)

Abiotic stresses usually cause protein function disorder in the ER, including protein aggregation, misfolding, and denatureation, and activate the unfolded protein response (UPR) [69]. Under normal conditions, ER organelle is the origin of initial protein synthesis. Hsp90 is required to play important roles in correct protein folding. When ER was subjected to diverse environments, such as drought, salt, and heat, the UPR was triggered. In higher eukaryotes, IRE-1(a approximately 100kDa type I transmembrane protein) contains Kinase and endoribonuclease domains, and is generally considered to be the most important signaling pathway from the ER [69]. Hsp90/IRE1 interaction might potentially cause the IRE1 response and affect the death of the cell during ER stress [69]. In our study, based on cellular location and evolution classification, *Bradi1g30130* in the *B*. *distachyon* was distributed in the ER (Figs 1 and 4). The evolutionary distribution of *Bradi1g30130* was similar to that of *At4g24190* (AtHsp90.7) and homologous (73%). The expression pattern of *Bradi1g30130* generally showed a significant up-regulated trend in response to drought and salt stresses, especially at 48h time points in the leaf tissue. *AtHsp90*.*7* was previously shown to be more sensitive than *AtHsp90*.*2* in response to drought and salt stresses [56]. Excessive Hsp90 in ER may reduce the shift and targeting of plasma membrane or vacuole membrane ion transporters to cope with salt stress by relieving cytosolic Na^+^[70]. Additionally, excessive Hsp90, especially in the chloroplast or ER, might trigger a general homeostasis itself, either by changing the organelle import/export system or the native protein homeostasis to enhance normal salt and osmotic stress tolerances [56]. These results suggest that the Hsp90 level in plant is critical for homeostasis of stress response and/or tolerance proteins.

## Conclusions

Based on the evolution analysis and molecular structure from 10 species representing four major plant lineages, the *Hsp90* gene family was found to be relatively conservative, and showed function diversity in the cellular location, gene duplication and evolution distribution between each group. Under drought and salt stresses, *BdHsp90* gene family in root and leaf tissue showed different expression pattern in each group, especially in *Bradi1g30130* and *Bradi3g3889700*, suggesting that they might be involved in different stress responses. Given the above mentioned findings and those published in the literatures, we suggest that *Hsp90* gene family plays roles in cytoplasm ABA signaling, ER stress protection and plant development under stress. Despite the detailed mechanism of BdHsp90 involvement in stress not being clearly understood, their function characterization will provide new insights into stress-responsive pathways.

## Supporting information

S1 Fig The number of Hsp90 genes in ten chosen species. Cr: *Chlamydomonas reinhardtii*, Pp: *Physcomitrella patens*, Bd: *Brachypodium distachyon*, Os: *Oryza sativa*, Ta: *Triticum aestivum*, Zm: *Zea mays*, At: *Arabidopsis thaliana*, Gm: *Glycine max*, Mt: *Medicago sativa*, Gr: *Gossypium raimondii*.
(TIF)

S2 Fig Molecular evolution clock of Hsp90 genes in ten chosen species. (TIF)

S3 Fig Standard curves and melting temperature curves of qRT-PCR. The blue standard curves represent the reference gene (ubiquitin) and the red standard curves represent the target genes.
(TIF)

S1 Table Primer sequences and relative expressions used for quantitative real-time RT-PCR in seven *Brachypodium distachyon* Hsp90 genes. (DOCX)

S2 Table Characteristics of the HSP90 genes and their deduced proteins in 10 representative species. (DOCX)

S3 Table The parameters of conserved Hsp90 domain from SMART and Pfam. (DOCX)

S4 Table Segmental duplicates and tandem duplicates of Hsp90 genes in ten plant species. (DOCX)

S5 Table Hsp90 gene subcellular localization results in each subgroup and species by TargetP1.1, WoLF PSORT and Predotar v. 1.03 on-line tools. (DOCX)

## Abbreviations

Hsp90: Heat shock proteins 90
BLAST: Basic local alignment search tool
ABA: Abscisic acid
ABI5: ABA insensitive five
CDS: Corresponding coding sequences
ER: endoplasmic reticulum
MCMC: Markov Chain Monte Carlo
UPS: Ubiquitin proteasome system
UPR: Unfolded protein response
MCMC: Markov Chain Monte Carlo
PGDD: Plant Genome Duplication Database
DREB2A: Dehydration-responsive element-binding protein 2A
RD22: The responsive of ehydration 22
Hsf: Heat shock factor
ABRE: ABA receptor element
DRE: Dehydration-responsive element
HSE: Heat shock element
qRT-PCR: Quantitative real-time polymerase chain reaction
