# Supplementary figures and images for: Genome-wide analysis of the Brachypodium distachyon (L.) P. Beauv. Hsp90 gene family reveals molecular evolution and expression profiling under drought and salt stresses
Source: PLoS One. 2017 Dec 7;12(12):e0189187. doi: 10.1371/journal.pone.0189187 (PMC5720741; doi:10.1371/journal.pone.0189187)

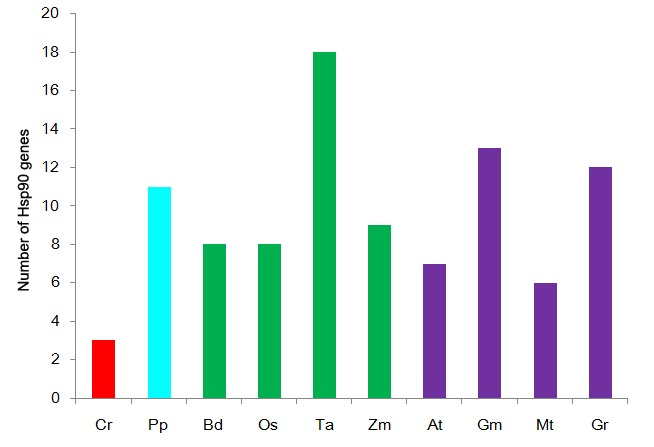

Supplement: S1 Fig — Cr: Chlamydomonas reinhardtii, Pp: Physcomitrella patens, Bd: Brachypodium distachyon, Os: Oryza sativa, Ta: Triticum aestivum, Zm: Zea mays, At: Arabidopsis thaliana, Gm: Glycine max, Mt: Medicago sativa, Gr: Gossypium raimondii. (TIF) [file pone.0189187.s001.tif]

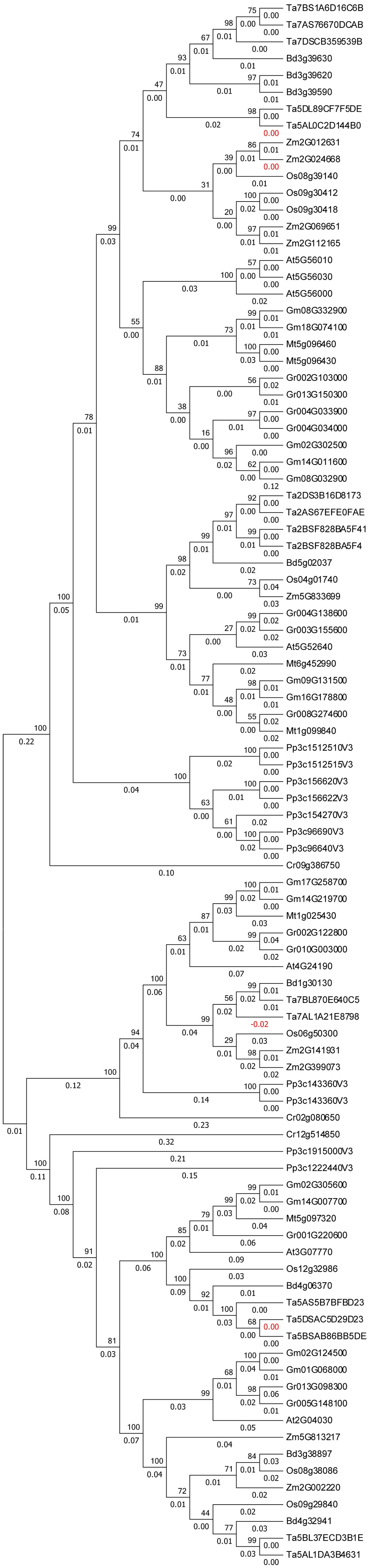

Supplement: S2 Fig — (TIF) [file pone.0189187.s002.tif]

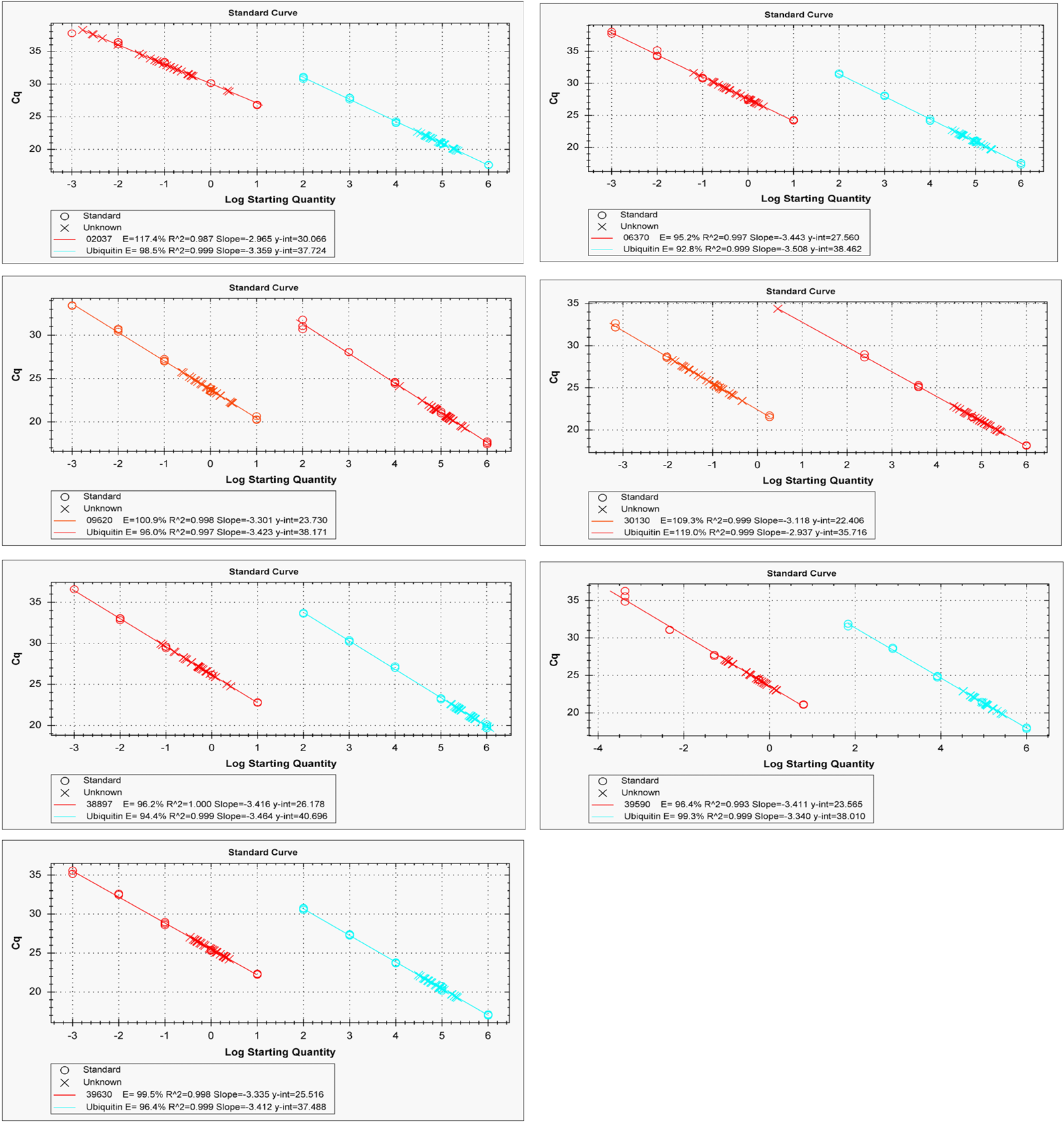

Supplement: S3 Fig — The blue standard curves represent the reference gene (ubiquitin) and the red standard curves represent the target genes. (TIF) [file pone.0189187.s003.tif]
