# Supplementary material for: Genome-wide analysis of the Brachypodium distachyon (L.) P. Beauv. Hsp90 gene family reveals molecular evolution and expression profiling under drought and salt stresses
Source: PLoS One. 2017 Dec 7;12(12):e0189187. doi: 10.1371/journal.pone.0189187 (PMC5720741; doi:10.1371/journal.pone.0189187)
Supplement: S1 Table — (DOCX) [file pone.0189187.s004.docx]

| **S1A Table. Primer sequences used for quantitative real-time RT-PCR in seven Hsp90 genes** | | | | | |
| --- | --- | --- | --- | --- | --- |
| **Gene name** | ***Primer name** | ***Primer sequence (5'to3')** | **Bp No.** | **Protein Length** |  |
| Bradi5g02037 | 02037-F | ACCCCATCTACCTCTGGACC | 20 | 710 |  |
|  | 02037-R | GGCTCACCTCCTTCACCTTC | 20 |  |  |
|  |  |  |  |  |  |
| Bradi3g39620 | 39620-F | CTGACAAGGCCACAAACACG | 20 | 699 |  |
|  | 39620-R | AGCCAACACCAAACTGACCA | 20 |  |  |
|  |  |  |  |  |  |
| Bradi3g39590 | 39590-F | CTGAGGAGGGCAAGGTTGAG | 20 | 700 |  |
|  | 39590-R | AGTCGTTGGTCAGGCTCTTG | 20 |  |  |
|  |  |  |  |  |  |
| Bradi3g39630 | 39630-F | ACGAACACACTCACGCTCAT | 20 | 699 |  |
|  | 39630-R | AGCCAACACCAAACTGACCA | 20 |  |  |
|  |  |  |  |  |  |
| Bradi1g30130 | 30130-F | TACCAGACGGCTCTCATGGA | 20 | 807 |  |
|  | 30130-R | CGCCTTTGTGGTCTCCTTCT | 20 |  |  |
|  |  |  |  |  |  |
| Bradi4g06370 | 06370-F | TCCCCGTGTGTTCTAGTTGC | 20 | 813 |  |
|  | 06370-R | TGGACTGTGCCCTCATCAAC | 20 |  |  |
|  |  |  |  |  |  |
| Bradi4g32941 | 32941-F | GTGGGACTTGGGGAAGAAGG | 20 | 794 |  |
|  | 32941-R | GCCTGCCAAACGTCCAAAAT | 20 |  |  |
|  |  |  | 0 |  |  |
| Bradi3g38897 | 38897-F | AGACCGCTCTGATCTCCAGT | 20 | 783 |  |
|  | 38897-R | AGACCTACCCCATCTCCCAC | 20 |  |  |

**S1B Table. Seven Hsp90 genes q-RT results under stress conditions**

| Target | Sample | Ctrl | Expression | Expression SEM | Corrected Expression SEM | Mean Cq | Cq SEM |
| --- | --- | --- | --- | --- | --- | --- | --- |
| 02037 | L-0 |  | 0.15249 | 0.01626 | 0.02045 | 33.25 | 0.13718 |
| 02037 | NL-12 |  | 0.00565 | 0.00143 | 0.00172 | 37.96 | 0.30883 |
| 02037 | NL-24 |  | 0.09245 | 0.00796 | 0.01039 | 32.77 | 0.10971 |
| 02037 | NL-48 |  | 0.18464 | 0.06230 | 0.06395 | 33.07 | 0.43325 |
| 02037 | NR-12 |  | 0.28533 | 0.01522 | 0.02419 | 32.43 | 0.00293 |
| 02037 | NR-24 |  | 0.16630 | 0.00341 | 0.01080 | 32.20 | 0.01365 |
| 02037 | NR-48 |  | 0.05337 | 0.00594 | 0.00769 | 33.79 | 0.13742 |
| 02037 | PL-12 |  | 0.01465 | 0.00134 | 0.00205 | 34.53 | 0.10964 |
| 02037 | PL-24 |  | 0.00799 | 0.00493 | 0.00507 | 36.79 | 0.79392 |
| 02037 | PL-48 |  | 0.00609 | 0.00004 | 0.00131 | 37.01 | 0.00000 |
| 02037 | PR-12 |  | 1.01045 | 0.12269 | 0.12269 | 28.94 | 0.06138 |
| 02037 | PR-24 |  | 0.32941 | 0.04386 | 0.04639 | 31.37 | 0.16872 |
| 02037 | PR-48 |  | 0.28341 | 0.00836 | 0.01519 | 31.31 | 0.02684 |
| 02037 | R-0 |  | 0.11592 | 0.02063 | 0.02151 | 31.71 | 0.22587 |
| Ubiquitin | L-0 |  |  |  |  | 22.07 | 0.00589 |
| Ubiquitin | NL-12 |  |  |  |  | 22.60 | 0.11736 |
| Ubiquitin | NL-24 |  |  |  |  | 20.80 | 0.01766 |
| Ubiquitin | NL-48 |  |  |  |  | 22.15 | 0.03750 |
| Ubiquitin | NR-12 |  |  |  |  | 22.05 | 0.07775 |
| Ubiquitin | NR-24 |  |  |  |  | 21.01 | 0.02560 |
| Ubiquitin | NR-48 |  |  |  |  | 21.16 | 0.04585 |
| Ubiquitin | PL-12 |  |  |  |  | 20.11 | 0.04956 |
| Ubiquitin | PL-24 |  |  |  |  | 21.79 | 0.03324 |
| Ubiquitin | PL-48 |  |  |  |  | 21.63 | 0.00686 |
| Ubiquitin | PR-12 |  |  |  |  | 19.95 | 0.16293 |
| Ubiquitin | PR-24 |  |  |  |  | 21.06 | 0.03465 |
| Ubiquitin | PR-48 |  |  |  |  | 20.78 | 0.03044 |
| Ubiquitin | R-0 |  |  |  |  | 19.93 | 0.04395 |

| Target | Sample | Ctrl | Expression | Expression SEM | Corrected Expression SEM | Mean Cq | Cq SEM |
| --- | --- | --- | --- | --- | --- | --- | --- |
| 06370 | L-0 |  | 0.46376 | 0.04052 | 0.04211 | 30.13 | 0.01065 |
| 06370 | NL-12 |  | 0.28382 | 0.04371 | 0.04479 | 31.47 | 0.20514 |
| 06370 | NL-24 |  | 0.39224 | 0.01188 | 0.01386 | 29.23 | 0.03387 |
| 06370 | NL-48 |  | 0.46572 | 0.02735 | 0.02985 | 30.26 | 0.04225 |
| 06370 | NR-12 |  | 0.61777 | 0.05999 | 0.06140 | 29.65 | 0.14247 |
| 06370 | NR-24 |  | 0.74863 | 0.04550 | 0.04645 | 28.45 | 0.04427 |
| 06370 | NR-48 |  | 0.56753 | 0.02131 | 0.02321 | 28.96 | 0.02528 |
| 06370 | PL-12 |  | 0.84342 | 0.04801 | 0.04823 | 27.48 | 0.02964 |
| 06370 | PL-24 |  | 1.65655 | 0.20184 | 0.20239 | 27.98 | 0.14649 |
| 06370 | PL-48 |  | 0.77277 | 0.00574 | 0.01592 | 29.37 | 0.00414 |
| 06370 | PR-12 |  | 1.00000 | 0.22406 | 0.22406 | 26.74 | 0.33503 |
| 06370 | PR-24 |  | 2.02587 | 0.06438 | 0.06449 | 27.00 | 0.03707 |
| 06370 | PR-48 |  | 1.22375 | 0.11827 | 0.11840 | 27.36 | 0.01513 |
| 06370 | R-0 |  | 0.94904 | 0.04647 | 0.04647 | 26.82 | 0.06267 |
| Ubiquitin | L-0 |  |  |  |  | 21.99 | 0.13265 |
| Ubiquitin | NL-12 |  |  |  |  | 22.61 | 0.10659 |
| Ubiquitin | NL-24 |  |  |  |  | 20.82 | 0.03064 |
| Ubiquitin | NL-48 |  |  |  |  | 22.13 | 0.07842 |
| Ubiquitin | NR-12 |  |  |  |  | 21.93 | 0.02852 |
| Ubiquitin | NR-24 |  |  |  |  | 21.01 | 0.08086 |
| Ubiquitin | NR-48 |  |  |  |  | 21.11 | 0.05107 |
| Ubiquitin | PL-12 |  |  |  |  | 20.20 | 0.08129 |
| Ubiquitin | PL-24 |  |  |  |  | 21.74 | 0.11036 |
| Ubiquitin | PL-48 |  |  |  |  | 22.00 | 0.01049 |
| Ubiquitin | PR-12 |  |  |  |  | 19.71 | 0.00049 |
| Ubiquitin | PR-24 |  |  |  |  | 21.04 | 0.03029 |
| Ubiquitin | PR-48 |  |  |  |  | 20.65 | 0.14642 |
| Ubiquitin | R-0 |  |  |  |  | 19.71 | 0.03856 |

| Target | Sample | Ctrl | Expression | Expression SEM | Corrected Expression SEM | Mean Cq | Cq SEM |
| --- | --- | --- | --- | --- | --- | --- | --- |
| 39620 | L-0 |  | 1.16617 | 0.04841 | 0.05019 | 23.99 | 0.03096 |
| 39620 | NL-12 |  | 0.67274 | 0.02394 | 0.02819 | 25.72 | 0.05094 |
| 39620 | NL-24 |  | 0.77464 | 0.06731 | 0.06767 | 23.62 | 0.06779 |
| 39620 | NL-48 |  | 1.32602 | 1.05174 | 1.05204 | 25.23 | 0.00089 |
| 39620 | NR-12 |  | 1.08686 | 0.13947 | 0.14027 | 24.37 | 0.04356 |
| 39620 | NR-24 |  | 0.88773 | 0.06150 | 0.06203 | 23.63 | 0.09718 |
| 39620 | NR-48 |  | 0.73201 | 0.03558 | 0.03628 | 23.73 | 0.03803 |
| 39620 | PL-12 |  | 0.77978 | 0.00718 | 0.00901 | 23.29 | 0.00169 |
| 39620 | PL-24 |  | 0.66686 | 0.05230 | 0.05338 | 24.74 | 0.10585 |
| 39620 | PL-48 |  | 0.63961 | 0.10783 | 0.10839 | 24.92 | 0.14751 |
| 39620 | PR-12 |  | 0.92679 | 0.07017 | 0.07018 | 22.28 | 0.03819 |
| 39620 | PR-24 |  | 1.10443 | 0.04956 | 0.04990 | 23.02 | 0.00998 |
| 39620 | PR-48 |  | 1.23363 | 0.06451 | 0.06484 | 23.03 | 0.00021 |
| 39620 | R-0 |  | 1.12245 | 0.03764 | 0.03764 | 22.17 | 0.01622 |
| Ubiquitin | L-0 |  |  |  |  | 21.48 | 0.05271 |
| Ubiquitin | NL-12 |  |  |  |  | 22.45 | 0.00288 |
| Ubiquitin | NL-24 |  |  |  |  | 20.49 | 0.10837 |
| Ubiquitin | NL-48 |  |  |  |  | 22.95 | 1.17907 |
| Ubiquitin | NR-12 |  |  |  |  | 21.77 | 0.18534 |
| Ubiquitin | NR-24 |  |  |  |  | 20.70 | 0.02126 |
| Ubiquitin | NR-48 |  |  |  |  | 20.51 | 0.06054 |
| Ubiquitin | PL-12 |  |  |  |  | 20.16 | 0.01358 |
| Ubiquitin | PL-24 |  |  |  |  | 21.42 | 0.03932 |
| Ubiquitin | PL-48 |  |  |  |  | 21.55 | 0.19853 |
| Ubiquitin | PR-12 |  |  |  |  | 19.37 | 0.10536 |
| Ubiquitin | PR-24 |  |  |  |  | 20.39 | 0.06589 |
| Ubiquitin | PR-48 |  |  |  |  | 20.57 | 0.07773 |
| Ubiquitin | R-0 |  |  |  |  | 19.54 | 0.04692 |

| Target | Sample | Ctrl | Expression | Expression SEM | Corrected Expression SEM | Mean Cq | Cq SEM |
| --- | --- | --- | --- | --- | --- | --- | --- |
| 30130 | L-0 |  | 2.58968 | 0.14831 | 0.15202 | 27.16 | 0.02297 |
| 30130 | NL-12 |  | 2.10279 | 0.20478 | 0.20777 | 27.59 | 0.07544 |
| 30130 | NL-24 |  | 1.36701 | 0.06492 | 0.06542 | 24.99 | 0.06211 |
| 30130 | NL-48 |  | 4.68620 | 0.63243 | 0.63596 | 28.22 | 0.08741 |
| 30130 | NR-12 |  | 2.03440 | 0.40127 | 0.40187 | 26.43 | 0.08919 |
| 30130 | NR-24 |  | 1.53169 | 0.06574 | 0.06659 | 25.33 | 0.05643 |
| 30130 | NR-48 |  | 3.61288 | 0.79541 | 0.79551 | 25.84 | 0.08922 |
| 30130 | PL-12 |  | 1.88379 | 0.45209 | 0.45216 | 25.13 | 0.31113 |
| 30130 | PL-24 |  | 2.70472 | 0.53999 | 0.54092 | 27.01 | 0.17639 |
| 30130 | PL-48 |  | 8.08414 | 2.34946 | 2.35003 | 27.50 | 0.08409 |
| 30130 | PR-12 |  | 2.95400 | 0.24356 | 0.24356 | 24.93 | 0.10526 |
| 30130 | PR-24 |  | 0.64131 | 0.03951 | 0.03981 | 24.29 | 0.08289 |
| 30130 | PR-48 |  | 0.00415 | 0.02262 | 0.02262 | 24.12 | 0.04377 |
| 30130 | R-0 |  | 0.84999 | 0.01434 | 0.01437 | 23.47 | 0.00681 |
| Ubiquitin | L-0 |  |  |  |  | 22.07 | 0.06977 |
| Ubiquitin | NL-12 |  |  |  |  | 22.75 | 0.10188 |
| Ubiquitin | NL-24 |  |  |  |  | 20.85 | 0.01565 |
| Ubiquitin | NL-48 |  |  |  |  | 22.32 | 0.15117 |
| Ubiquitin | NR-12 |  |  |  |  | 21.70 | 0.23715 |
| Ubiquitin | NR-24 |  |  |  |  | 21.02 | 0.01311 |
| Ubiquitin | NR-48 |  |  |  |  | 20.41 | 0.26795 |
| Ubiquitin | PL-12 |  |  |  |  | 20.56 | 0.08826 |
| Ubiquitin | PL-24 |  |  |  |  | 21.88 | 0.19296 |
| Ubiquitin | PL-48 |  |  |  |  | 20.94 | 0.36214 |
| Ubiquitin | PR-12 |  |  |  |  | 19.81 | 0.03504 |
| Ubiquitin | PR-24 |  |  |  |  | 21.15 | 0.00880 |
| Ubiquitin | PR-48 |  |  |  |  | 27.42 | 6.95916 |
| Ubiquitin | R-0 |  |  |  |  | 20.02 | 0.02054 |

| Target | Sample | Ctrl | Expression | Expression SEM | Corrected Expression SEM | Mean Cq | Cq SEM |
| --- | --- | --- | --- | --- | --- | --- | --- |
| 38897 | L-0 |  | 0.68241 | 0.02103 | 0.02180 | 27.69 | 0.00608 |
| 38897 | NL-12 |  | 0.22549 | 0.01126 | 0.01176 | 29.89 | 0.06916 |
| 38897 | NL-24 |  | 0.74044 | 0.02317 | 0.02347 | 26.59 | 0.02691 |
| 38897 | NL-48 |  | 1.32665 | 0.13616 | 0.13641 | 26.96 | 0.14362 |
| 38897 | NR-12 |  | 0.30559 | 0.01669 | 0.01711 | 28.95 | 0.03655 |
| 38897 | NR-24 |  | 0.26855 | 0.02202 | 0.02218 | 28.17 | 0.12164 |
| 38897 | NR-48 |  | 0.26009 | 0.02596 | 0.02609 | 28.21 | 0.06836 |
| 38897 | PL-12 |  | 0.54080 | 0.13445 | 0.13448 | 26.60 | 0.30845 |
| 38897 | PL-24 |  | 3.90998 | 0.37116 | 0.37116 | 24.91 | 0.13926 |
| 38897 | PL-48 |  | 2.20179 | 0.19332 | 0.19345 | 25.98 | 0.08605 |
| 38897 | PR-12 |  | 0.35916 | 0.03607 | 0.03610 | 26.43 | 0.12847 |
| 38897 | PR-24 |  | 0.52529 | 0.04799 | 0.04812 | 27.10 | 0.07372 |
| 38897 | PR-48 |  | 0.43723 | 0.00925 | 0.00971 | 27.13 | 0.00448 |
| 38897 | R-0 |  | 0.23846 | 0.00256 | 0.00305 | 27.21 | 0.01069 |
| Ubiquitin | L-0 |  |  |  |  | 22.03 | 0.04594 |
| Ubiquitin | NL-12 |  |  |  |  | 22.60 | 0.02693 |
| Ubiquitin | NL-24 |  |  |  |  | 21.04 | 0.03835 |
| Ubiquitin | NL-48 |  |  |  |  | 22.29 | 0.05133 |
| Ubiquitin | NR-12 |  |  |  |  | 22.11 | 0.07333 |
| Ubiquitin | NR-24 |  |  |  |  | 21.11 | 0.00294 |
| Ubiquitin | NR-48 |  |  |  |  | 21.11 | 0.13317 |
| Ubiquitin | PL-12 |  |  |  |  | 20.58 | 0.20509 |
| Ubiquitin | PL-24 |  |  |  |  | 21.84 | 0.02140 |
| Ubiquitin | PL-48 |  |  |  |  | 22.06 | 0.09917 |
| Ubiquitin | PR-12 |  |  |  |  | 19.78 | 0.07651 |
| Ubiquitin | PR-24 |  |  |  |  | 21.04 | 0.11534 |
| Ubiquitin | PR-48 |  |  |  |  | 20.79 | 0.03151 |
| Ubiquitin | R-0 |  |  |  |  | 19.96 | 0.01195 |

| Target | Sample | Ctrl | Expression | Expression SEM | Corrected Expression SEM | Mean Cq | Cq SEM |
| --- | --- | --- | --- | --- | --- | --- | --- |
| 39590 | L-0 |  | 1.11213 | 0.04368 | 0.05126 | 25.14 | 0.00428 |
| 39590 | NL-12 |  | 0.51962 | 0.02048 | 0.03155 | 27.07 | 0.05751 |
| 39590 | NL-24 |  | 0.55228 | 0.02241 | 0.02594 | 25.09 | 0.05238 |
| 39590 | NL-48 |  | 0.39461 | 0.02319 | 0.02904 | 26.91 | 0.08640 |
| 39590 | NR-12 |  | 0.93344 | 0.00905 | 0.02716 | 25.43 | 0.01116 |
| 39590 | NR-24 |  | 1.02216 | 0.05673 | 0.05945 | 24.55 | 0.06217 |
| 39590 | NR-48 |  | 0.72324 | 0.15977 | 0.16043 | 24.78 | 0.32518 |
| 39590 | PL-12 |  | 0.70615 | 0.03565 | 0.03700 | 24.25 | 0.07038 |
| 39590 | PL-24 |  | 0.44213 | 0.01369 | 0.02224 | 26.50 | 0.01364 |
| 39590 | PL-48 |  | 0.30966 | 0.01740 | 0.02224 | 26.94 | 0.07582 |
| 39590 | PR-12 |  | 0.88371 | 0.03655 | 0.03659 | 23.20 | 0.01272 |
| 39590 | PR-24 |  | 1.24170 | 0.07526 | 0.07643 | 23.96 | 0.08925 |
| 39590 | PR-48 |  | 1.22480 | 0.08639 | 0.08718 | 23.86 | 0.10438 |
| 39590 | R-0 |  | 1.15697 | 0.13019 | 0.13019 | 23.02 | 0.01615 |
| Ubiquitin | L-0 |  |  |  |  | 22.11 | 0.05681 |
| Ubiquitin | NL-12 |  |  |  |  | 22.90 | 0.00985 |
| Ubiquitin | NL-24 |  |  |  |  | 21.05 | 0.02889 |
| Ubiquitin | NL-48 |  |  |  |  | 22.34 | 0.01046 |
| Ubiquitin | NR-12 |  |  |  |  | 22.14 | 0.00885 |
| Ubiquitin | NR-24 |  |  |  |  | 21.41 | 0.05267 |
| Ubiquitin | NR-48 |  |  |  |  | 21.14 | 0.03561 |
| Ubiquitin | PL-12 |  |  |  |  | 20.58 | 0.02477 |
| Ubiquitin | PL-24 |  |  |  |  | 22.11 | 0.04287 |
| Ubiquitin | PL-48 |  |  |  |  | 22.03 | 0.03360 |
| Ubiquitin | PR-12 |  |  |  |  | 19.88 | 0.05868 |
| Ubiquitin | PR-24 |  |  |  |  | 21.12 | 0.00945 |
| Ubiquitin | PR-48 |  |  |  |  | 21.00 | 0.00436 |
| Ubiquitin | R-0 |  |  |  |  | 20.09 | 0.16245 |

| Target | Sample | Ctrl | Expression | Expression SEM | Corrected Expression SEM | Mean Cq | Cq SEM |
| --- | --- | --- | --- | --- | --- | --- | --- |
| 39630 | L-0 |  | 1.20879 | 0.07607 | 0.07731 | 26.00 | 0.04964 |
| 39630 | NL-12 |  | 1.12484 | 0.14319 | 0.14449 | 26.86 | 0.17221 |
| 39630 | NL-24 |  | 2.45637 | 0.47083 | 0.47083 | 24.32 | 0.11604 |
| 39630 | NL-48 |  | 1.25230 | 0.11733 | 0.11860 | 26.35 | 0.12763 |
| 39630 | NR-12 |  | 0.91147 | 0.14567 | 0.14632 | 26.55 | 0.23137 |
| 39630 | NR-24 |  | 1.07673 | 0.11099 | 0.11125 | 25.37 | 0.07627 |
| 39630 | NR-48 |  | 0.47503 | 0.02114 | 0.02238 | 26.60 | 0.06014 |
| 39630 | PL-12 |  | 0.58475 | 0.03651 | 0.03678 | 25.45 | 0.08925 |
| 39630 | PL-24 |  | 1.46746 | 0.04990 | 0.05148 | 25.60 | 0.03833 |
| 39630 | PL-48 |  | 1.09577 | 0.01978 | 0.02557 | 26.50 | 0.00948 |
| 39630 | PR-12 |  | 0.91933 | 0.03758 | 0.03759 | 24.44 | 0.02700 |
| 39630 | PR-24 |  | 1.48562 | 0.12737 | 0.12762 | 25.11 | 0.02642 |
| 39630 | PR-48 |  | 1.59833 | 0.07668 | 0.07674 | 24.61 | 0.03297 |
| 39630 | R-0 |  | 0.81598 | 0.08194 | 0.08197 | 24.72 | 0.12845 |
| Ubiquitin | L-0 |  |  |  |  | 21.33 | 0.07820 |
| Ubiquitin | NL-12 |  |  |  |  | 22.10 | 0.06738 |
| Ubiquitin | NL-24 |  |  |  |  | 20.67 | 0.25800 |
| Ubiquitin | NL-48 |  |  |  |  | 21.75 | 0.04717 |
| Ubiquitin | NR-12 |  |  |  |  | 21.47 | 0.00735 |
| Ubiquitin | NR-24 |  |  |  |  | 20.51 | 0.13130 |
| Ubiquitin | NR-48 |  |  |  |  | 20.56 | 0.02369 |
| Ubiquitin | PL-12 |  |  |  |  | 19.70 | 0.01488 |
| Ubiquitin | PL-24 |  |  |  |  | 21.21 | 0.03163 |
| Ubiquitin | PL-48 |  |  |  |  | 21.70 | 0.02493 |
| Ubiquitin | PR-12 |  |  |  |  | 19.33 | 0.05391 |
| Ubiquitin | PR-24 |  |  |  |  | 20.73 | 0.12413 |
| Ubiquitin | PR-48 |  |  |  |  | 20.32 | 0.06257 |
| Ubiquitin | R-0 |  |  |  |  | 19.44 | 0.06979 |
