# Supplementary material for: Genome-wide analysis of the Brachypodium distachyon (L.) P. Beauv. Hsp90 gene family reveals molecular evolution and expression profiling under drought and salt stresses
Source: PLoS One. 2017 Dec 7;12(12):e0189187. doi: 10.1371/journal.pone.0189187 (PMC5720741; doi:10.1371/journal.pone.0189187)
Supplement: S2 Table — (DOCX) [file pone.0189187.s005.docx]

| **S2 Table. Characteristics of the HSP90 genes and their deduced proteins in 10 representative species** | | | | | | | | | |  |  |  |
| --- | --- | --- | --- | --- | --- | --- | --- | --- | --- | --- | --- | --- |
| **Lineage** | **Organism** | **Nomenclature** | **Gene locus** | **Chr.** | **Locations on Chr.** | **Group** | **Genomics Length** | **CDS Length** | **Protein Length** | **No. of Introns** | **PI** | **MW** |
| **Algae** | ***Chlamydomonas reinhardtii*** | ***Cr09g386750*** | ***Cre09.g386750*** | **Chr 9** | **264243-268808** | **Ⅰ** | **4566** | **2118** | **705** | ***7*** | **4.99** | **80681.54** |
|  |  | ***Cr12g514850*** | ***Cre12.g514850*** | **Chr 12** | **3726829-3732140** | **Ⅲ** | **5312** | **4233** | **810** | ***8*** | **5.24** | **89353.72** |
|  |  | ***Cr02g080650*** | ***Cre02.g080650*** | **Chr 02** | **1040659-1047025** | **Ⅱ** | **6367** | **2460** | **819** | ***11*** | **4.83** | **93119.79** |
| **Mosses** | ***Physcomitrella patens*** | ***Pp3c156620V3*** | ***Pp3c15_6620*** | **Chr 15** | **4067973-4071200** | **Ⅰ** | **3228** | **2115** | **704** | ***1*** | **5.04** | **80741.71** |
|  |  | ***Pp3c156622V3*** | ***Pp3c15_6622*** | **Chr 15** | **4068065-4071127** | **Ⅰ** | **3063** | **2115** | **704** | ***1*** | **5.04** | **80741.71** |
|  |  | ***Pp3c1512510V3*** | ***Pp3c15_12510*** | **Chr 15** | **8294108-8297217** | **Ⅰ** | **3110** | **2109** | **702** | ***1*** | **5.06** | **80305.24** |
|  |  | ***Pp3c1512515V3*** | ***Pp3c15_12515*** | **Chr 12** | **8294102-8297562** | **Ⅰ** | **3461** | **2109** | **702** | ***1*** | **5.06** | **80305.24** |
|  |  | ***Pp3c96690V3*** | ***Pp3c9_6690*** | **Chr 9** | **3810723-3813891** | **Ⅰ** | **3169** | **2115** | **704** | ***1*** | **5.02** | **80858.76** |
|  |  | ***Pp3c96640V3*** | ***Pp3c9_6640*** | **Chr 9** | **3792759-3795809** | **Ⅰ** | **3051** | **2124** | **707** | ***1*** | **5.02** | **81212.23** |
|  |  | ***Pp3c154270V3*** | ***Pp3c15_4270*** | **Chr 15** | **2714924-2717836** | **Ⅰ** | **2913** | **2106** | **701** | ***1*** | **4.99** | **80456.16** |
|  |  | ***Pp3c143360V3*** | ***Pp3c15_3360*** | **Chr 14** | **2281348-2288235** | **Ⅱ** | **6888** | **2550** | **849** | ***16*** | **4.87** | **96837.80** |
|  |  | ***Pp3c4810V3*** | ***Pp3c4_810*** | **Chr 04** | **456254-462741** | **Ⅲ** | **6488** | **2415** | **804** | ***17*** | **5.15** | **91086.28** |
|  |  | ***Pp3c1222440V3*** | ***Pp3c12_22440*** | **Chr 12** | **14850088-14856595** | **Ⅲ** | **6508** | **2436** | **811** | ***17*** | **5.15** | **91482.58** |
|  |  | ***Pp3c1915000V3*** | ***Pp3c19_15000*** | **Chr 19** | **9899296-9906739** | **Ⅲ** | **7444** | **2421** | **806** | ***19*** | **5.89** | **91270.91** |
| **Monocots** | ***Brachypodium distachyon*** | ***Bd5g02037*** | ***Bradi5g02037*** | **Chr 05** | **2168828-2171872** | **Ⅰ** | **3045** | **2013** | **710** | ***2*** | **4.97** | **81166.10** |
|  |  | ***Bd3g39620*** | ***Bradi3g39620*** | **Chr 03** | **41591254-41595894** | **Ⅰ** | **4641** | **2100** | **699** | ***2*** | **4.95** | **80176.88** |
|  |  | ***Bd3g39590*** | ***Bradi3g39590*** | **Chr 03** | **41576531-41580838** | **Ⅰ** | **4308** | **2103** | **700** | ***2*** | **4.96** | **80389.21** |
|  |  | ***Bd3g39630*** | ***Bradi3g39630*** | **Chr 03** | **41610449-41615461** | **Ⅰ** | **5013** | **2100** | **699** | ***2*** | **4.93** | **80253.91** |
|  |  | ***Bd1g30130*** | ***Bradi1g30130*** | **Chr 01** | **25571488-25577219** | **Ⅱ** | **5732** | **2424** | **807** | ***14*** | **4.91** | **92886.53** |
|  |  | ***Bd4g06370*** | ***Bradi4g06370*** | **Chr 04** | **5330059-5336369** | **Ⅲ** | **6311** | **2442** | **813** | ***19*** | **5.20** | **91321.00** |
|  |  | ***Bd4g32941*** | ***Bradi4g32941*** | **Chr 04** | **38635797-38640581** | **Ⅲ** | **4785** | **2385** | **794** | ***18*** | **5.10** | **89544.37** |
|  |  | ***Bd3g38897*** | ***Bradi3g38897*** | **Chr 03** | **41105766-41110850** | **Ⅲ** | **5085** | **2392** | **783** | ***18*** | **4.91** | **88520.93** |
|  | ***Oryza sativa*** | ***Os04g01740*** | ***LOC_Os04g01740*** | **Chr 04** | **483241-486065** | **Ⅰ** | **2825** | **2112** | **703** | ***2*** | **5.04** | **80250.22** |
|  |  | ***Os08g39140*** | ***LOC_Os08g39140*** | **Chr 08** | **24719086-24723553** | **Ⅰ** | **4468** | **2100** | **699** | ***2*** | **4.99** | **80194.04** |
|  |  | ***Os09g30412*** | ***LOC_Os09g30412*** | **Chr 09** | **18514572-18518316** | **Ⅰ** | **3745** | **2100** | **699** | ***2*** | **4.97** | **80199.98** |
|  |  | ***Os09g30418*** | ***LOC_Os09g30418*** | **Chr 09** | **18535746-18541109** | **Ⅰ** | **5364** | **2493** | **830** | ***3*** | **5.15** | **94195.46** |
|  |  | ***Os09g29840*** | ***LOC_Os09g29840*** | **Chr 09** | **18150618-18155512** | **Ⅲ** | **4895** | **2376** | **791** | ***18*** | **5.03** | **89219.85** |
|  |  | ***Os08g38086*** | ***LOC_Os08g38086*** | **Chr 08** | **24124838-24129488** | **Ⅲ** | **4651** | **2286** | **761** | ***19*** | **4.97** | **85878.03** |
|  |  | ***Os12g32986*** | ***LOC_Os12g32986*** | **Chr 12** | **19921576-19927766** | **Ⅲ** | **6191** | **2436** | **811** | ***19*** | **5.21** | **91480.23** |
|  |  | ***Os06g50300*** | ***LOC_Os06g50300*** | **Chr 06** | **30444411-30450497** | **Ⅱ** | **6037** | **2439** | **812** | ***14*** | **4.89** | **93044.79** |
|  | ***Triticum aestivum*** | ***Ta2DS3B16D8173*** | ***Traes_2DS_3B16D8173*** | **Chr 2DS** | **8613-11951** | **Ⅰ** | **3339** | **2139** | **712** | ***2*** | **4.93** | **81445.22** |
|  |  | ***Ta2AS67EFE0FAE*** | ***Traes_2AS_67EFE0FAE*** | **Chr 2AS** | **7053-10425** | **Ⅰ** | **3373** | **2487** | **828** | ***2*** | **5.53** | **94182.74** |
|  |  | ***Ta2BSF828BA5F41*** | ***Traes_2BS_F828BA5F41*** | **Chr 2BS** | **14331-16804** | **Ⅰ** | **2474** | **2139** | **712** | ***2*** | **4.94** | **81290.03** |
|  |  | ***Ta2BSF828BA5F4*** | ***Traes_2BS_F828BA5F4*** | **Chr2BS** | **22862-25335** | **Ⅰ** | **2474** | **2139** | **712** | ***2*** | **4.94** | **81290.03** |
|  |  | ***Ta7BS1A6D16C6B*** | ***Traes_7BS_1A6D16C6B*** | **Chr 7BS** | **3474-7231** | **Ⅰ** | **3758** | **2103** | **700** | ***2*** | **4.95** | **79539.26** |
|  |  | ***Ta7DSCB359539B*** | ***Traes_7DS_CB359539B*** | **Chr 7DS** | **171-4142** | **Ⅰ** | **3972** | **2103** | **700** | ***2*** | **4.93** | **69970.74** |
|  |  | ***Ta5DL89CF7F5DE*** | ***Traes_5DL_89CF7F5DE*** | **Chr 5DL** | **4990-8721** | **Ⅰ** | **3732** | **2103** | **700** | ***2*** | **4.95** | **80460.28** |
|  |  | ***Ta5BL0F3A986F9*** | ***Traes_5BL_0F3A986F9*** | **Chr 5BL** | **6515-10786** | **Ⅰ** | **4272** | **2103** | **700** | ***2*** | **4.95** | **80460.28** |
|  |  | ***Ta7AS76670DCAB*** | ***Traes_7AS_76670DCAB*** | **Chr 7AS** | **3504-6448** | **Ⅰ** | **2945** | **1848** | **615** | ***1*** | **4.83** | **79264.88** |
|  |  | ***Ta5AL0C2D144B0*** | ***Traes_5AL_0C2D144B0*** | **Chr 5AL** | **8251-11487** | **Ⅰ** | **3237** | **1848** | **615** | ***1*** | **5.02** | **70927.46** |
|  |  | ***Ta5BL37ECD3B1E*** | ***Traes_5BL_37ECD3B1E*** | **Chr 5BL** | **18-4193** | **Ⅲ** | **4176** | **2088** | **695** | ***17*** | **4.80** | **79421.68** |
|  |  | ***Ta5DL5A546D5B3*** | ***Traes_5DL_5A546D5B3*** | **Chr 5DL** | **129-4324** | **Ⅲ** | **4196** | **2088** | **695** | ***17*** | **4.80** | **79435.70** |
|  |  | ***Ta5AS5B7BFBD23*** | ***Traes_5AS_5B7BFBD23*** | **Chr 5AS** | **2-5978** | **Ⅲ** | **5977** | **2085** | **694** | ***17*** | **4.98** | **79293.67** |
|  |  | ***Ta7BL870E640C5*** | ***Traes_7BL_870E640C5*** | **Chr 7BL** | **264-11230** | **Ⅱ** | **10967** | **2151** | **716** | ***12*** | **4.84** | **82475.73** |
|  |  | ***Ta5AL1DA3B4631*** | ***Traes_5AL_1DA3B4631*** | **Chr 5AL** | **1061-5102** | **Ⅲ** | **4042** | **2088** | **695** | ***17*** | **4.81** | **79482.76** |
|  |  | ***Ta5DSAC5D29D23*** | ***Traes_5DS_AC5D29D23*** | **Chr 5DS** | **4413-10712** | **Ⅲ** | **6300** | **2442** | **813** | ***19*** | **4.85** | **74389.58** |
|  |  | ***Ta7AL1A21E8798*** | ***Traes_7AL_1A21E8798*** | **Chr 7AL** | **1-3554** | **Ⅱ** | **3554** | **641** | **547** | ***11*** | **4.92** | **63993.24** |
|  |  | ***Ta5BSAB86BB5DE*** | ***Traes_5BS_AB86BB5DE*** | **Chr 5BS** | **8803-15114** | **Ⅲ** | **6312** | **2448** | **815** | ***16*** | **5.05** | **79482.76** |
|  | ***Zea mays*** | ***Zm5G833699*** | ***GRMZM5G833699*** | **Chr 10** | **93697766-93700740** | **Ⅰ** | **3172** | **837** | **278** | ***1*** | **5.03** | **81802.65** |
|  |  | ***Zm2G069651*** | ***GRMZM2G069651*** | **Chr 07** | **131816486-131820321** | **Ⅰ** | **3836** | **2100** | **699** | ***2*** | **5.04** | **80274.18** |
|  |  | ***Zm2G112165*** | ***GRMZM2G112165*** | **Chr 02** | **190093038-190097209** | **Ⅰ** | **4172** | **2097** | **698** | ***2*** | **5.01** | **80172.04** |
|  |  | ***Zm2G012631*** | ***GRMZM2G012631*** | **Chr 04** | **198547817-198551900** | **Ⅰ** | **4084** | **2100** | **699** | ***2*** | **4.97** | **80362.22** |
|  |  | ***Zm2G141931*** | ***GRMZM2G141931*** | **Chr 06** | **83941625-83947219** | **Ⅱ** | **5595** | **2415** | **804** | ***14*** | **4.95** | **92572.23** |
|  |  | ***Zm2G399073*** | ***GRMZM2G399073*** | **Chr 05** | **61555816-61563290** | **Ⅱ** | **7475** | **3006** | **1001** | ***16*** | **4.99** | **113162.49** |
|  |  | ***Zm2G024668*** | ***GRMZM2G024668*** | **Chr 01** | **184272689-184277348** | **Ⅰ** | **4660** | **1056** | **351** | ***2*** | **4.98** | **40598.94** |
|  |  | ***Zm2G002220*** | ***GRMZM2G002220*** | **Chr 04** | **197541805-197546387** | **Ⅲ** | **4583** | **2382** | **793** | ***18*** | **5.04** | **89407.18** |
|  |  | ***Zm5G813217*** | ***GRMZM5G813217*** | **Chr 07** | **129998945-130003537** | **Ⅲ** | **4593** | **2277** | **758** | ***17*** | **5.11** | **86128.69** |
| **Dicots** | ***Arabidopsis thaliana*** | ***At5G52640*** | ***AT5G52640*** | **Chr 05** | **21352399-21355361** | **Ⅰ** | **2693** | **2118** | **705** | ***3*** | **4.95** | **81180.09** |
|  |  | ***At5G56000*** | ***AT5G56000*** | **Chr 05** | **22677451-22680152** | **Ⅰ** | **2702** | **2100** | **699** | ***2*** | **4.96** | **80141.15** |
|  |  | ***At5G56010*** | ***AT5G56010*** | **Chr 05** | **22681305-22684129** | **Ⅰ** | **2825** | **2100** | **699** | ***2*** | **4.95** | **80052.07** |
|  |  | ***At5G56030*** | ***AT5G56030*** | **Chr 05** | **22686802-22689650** | **Ⅰ** | **2849** | **2100** | **699** | ***2*** | **4.94** | **83126.64** |
|  |  | ***At4G24190*** | ***AT4G24190*** | **Chr 04** | **12551717-12555909** | **Ⅱ** | **4193** | **2475** | **823** | ***14*** | **4.94** | **94204.01** |
|  |  | ***At2G04030*** | ***AT2G04030*** | **Chr 02** | **1281841-1286104** | **Ⅲ** | **4264** | **2343** | **780** | ***18*** | **4.93** | **88662.95** |
|  |  | ***At3G07770*** | ***AT3G07770*** | **Chr 03** | **2479548-2484194** | **Ⅲ** | **4647** | **2400** | **799** | ***19*** | **5.26** | **90566.86** |
|  | ***Glycine max*** | ***Gm09G131500*** | ***Glyma.09G131500*** | **Chr 09** | **32754805-32758309** | **Ⅰ** | **3505** | **2100** | **699** | ***3*** | **4.95** | **80389.25** |
|  |  | ***Gm16G178800*** | ***Glyma.16G178800*** | **Chr 16** | **33950003-33953968** | **Ⅰ** | **3966** | **2100** | **699** | ***3*** | **4.97** | **80285.13** |
|  |  | ***Gm08G332900*** | ***Glyma.08G332900*** | **Chr 08** | **44997823-45000952** | **Ⅰ** | **3130** | **2100** | **699** | ***1*** | **4.97** | **80184.19** |
|  |  | ***Gm14G011600*** | ***Glyma.14G011600*** | **Chr 14** | **887156-890657** | **Ⅰ** | **3502** | **2103** | **700** | ***2*** | **4.98** | **80187.10** |
|  |  | ***Gm18G074100*** | ***Glyma.18G074100*** | **Chr 08** | **7029009-7032141** | **Ⅰ** | **3133** | **2109** | **702** | ***2*** | **4.94** | **80380.34** |
|  |  | ***Gm02G302500*** | ***Glyma.02G302500*** | **Chr 02** | **47792409..47795587** | **Ⅰ** | **3179** | **2109** | **702** | ***2*** | **4.96** | **80329.26** |
|  |  | ***Gm08G032900*** | ***Glyma.08G032900*** | **Chr 08** | **2620906-2623443** | **Ⅰ** | **2538** | **1968** | **655** | ***2*** | **6.28** | **75877.28** |
|  |  | ***Gm17G258700*** | ***Glyma.17G258700*** | **Chr 17** | **41280124-41284980** | **Ⅱ** | **4857** | **2445** | **814** | ***14*** | **4.86** | **93293.63** |
|  |  | ***Gm14G219700*** | ***Glyma.14G219700*** | **Chr 14** | **48476500-48481440** | **Ⅱ** | **4941** | **2544** | **847** | ***13*** | **4.91** | **97379.42** |
|  |  | ***Gm02G124500*** | ***Glyma.02G124500*** | **Chr 02** | **12445411-12453687** | **Ⅲ** | **8277** | **2345** | **794** | ***18*** | **4.91** | **90091.55** |
|  |  | ***Gm02G305600*** | ***Glyma.02G305600*** | **Chr 02** | **48034339-48039586** | **Ⅲ** | **5248** | **3276** | **791** | ***18*** | **5.25** | **89716.27** |
|  |  | ***Gm01G068000*** | ***Glyma.01G068000*** | **Chr 01** | **11288738-11297869** | **Ⅲ** | **9132** | **2382** | **793** | ***18*** | **4.94** | **90114.59** |
|  |  | ***Gm14G007700*** | ***Glyma.14G007700*** | **Chr 14** | **600842-606096** | **Ⅲ** | **5255** | **2394** | **797** | ***18*** | **5.13** | **90502.91** |
|  | ***Medicago truncatula*** | ***Mt6g452990*** | ***Medtr6g452990*** | **Chr 06** | **18619049-18622693** | **Ⅰ** | **3645** | **2100** | **699** | ***3*** | **4.96** | **80301.25** |
|  |  | ***Mt1g099840*** | ***Mt1g099840*** | **Chr 01** | **45060414-45063354** | **Ⅰ** | **2941** | **2070** | **689** | ***3*** | **4.96** | **79365.13** |
|  |  | ***Mt5g096460*** | ***Mt5g096460*** | **Chr 05** | **42174906-42178554** | **Ⅰ** | **3649** | **2100** | **699** | ***2*** | **4.95** | **80161.19** |
|  |  | ***Mt5g096430*** | ***Mt5g096430*** | **Chr 05** | **42164519-42167895** | **Ⅰ** | **3377** | **2100** | **699** | ***2*** | **4.95** | **80161.19** |
|  |  | ***Mt5g097320*** | ***Mt5g097320*** | **Chr 05** | **8126353-8131899** | **Ⅲ** | **6675** | **2379** | **792** | ***19*** | **5.34** | **90082.71** |
|  |  | ***Mt1g025430*** | ***Mt1g025430*** | **Chr 01** | **3274709-3275901** | **Ⅱ** | **5547** | **2457** | **818** | ***14*** | **4.79** | **94109.50** |
|  | ***Gossypium raimondii*** | ***Gr004G138600*** | ***Gorai.004G138600*** | **Chr 04** | **38870293-38873451** | **Ⅰ** | **3159** | **2115** | **704** | ***3*** | **5.06** | **81025.24** |
|  |  | ***Gr008G274600*** | ***Gorai.008G274600*** | **Chr 08** | **55290618-55294359** | **Ⅰ** | **3742** | **2112** | **703** | ***3*** | **5.00** | **80743.75** |
|  |  | ***Gr003G155600*** | ***Gorai.003G155600*** | **Chr 03** | **42463639-42467252** | **Ⅰ** | **3614** | **2124** | **707** | ***3*** | **5.06** | **81117.10** |
|  |  | ***Gr002G103000*** | ***Gorai.002G103000*** | **Chr 02** | **13118219-13121608** | **Ⅰ** | **3390** | **2100** | **699** | ***2*** | **4.98** | **80007.04** |
|  |  | ***Gr004G033900*** | ***Gorai.004G033900*** | **Chr 04** | **2773498-2776641** | **Ⅰ** | **3144** | **2100** | **699** | ***2*** | **5.00** | **80020.02** |
|  |  | ***Gr013G150300*** | ***Gorai.013G150300*** | **Chr 13** | **41037208-41040239** | **Ⅰ** | **3032** | **2100** | **699** | ***2*** | **5.00** | **80125.05** |
|  |  | ***Gr004G034000*** | ***Gorai.004G034000*** | **Chr 04** | **2786127-2789058** | **Ⅰ** | **2932** | **2001** | **666** | ***3*** | **5.04** | **76095.60** |
|  |  | ***Gr002G122800*** | ***Gorai.002G122800*** | **Chr 02** | **17649893-17654527** | **Ⅱ** | **4635** | **2430** | **809** | ***14*** | **4.94** | **92406.96** |
|  |  | ***Gr001G220600*** | ***Gorai.001G220600*** | **Chr 09** | **44749309-44754987** | **Ⅲ** | **5679** | **2394** | **794** | ***19*** | **5.25** | **90447.96** |
|  |  | ***Gr013G098300*** | ***Gorai.013G098300*** | **Chr 13** | **17479456-17484611** | **Ⅲ** | **5156** | **2334** | **777** | ***19*** | **4.92** | **89035.88** |
|  |  | ***Gr005G148100*** | ***Gorai.005G148100*** | **Chr 05** | **40579047-40585315** | **Ⅲ** | **6269** | **2499** | **832** | ***17*** | **4.87** | **94919.47** |
|  |  | ***Gr010G003000*** | ***Gorai.010G003000*** | **Chr 10** | **124969-135837** | **Ⅱ** | **10869** | **3255** | **1084** | ***21*** | **5.08** | **123601.3** |
