# Supplementary material for: Genome-wide analysis of the Brachypodium distachyon (L.) P. Beauv. Hsp90 gene family reveals molecular evolution and expression profiling under drought and salt stresses
Source: PLoS One. 2017 Dec 7;12(12):e0189187. doi: 10.1371/journal.pone.0189187 (PMC5720741; doi:10.1371/journal.pone.0189187)
Supplement: S3 Table — (DOCX) [file pone.0189187.s006.docx]

| **S3Table. The parameters of conserved Hsp90 domain from SMART and Pfam.**  **A. The parameters of conserved Hsp90 domain from SMART** | | | | | | | | | | |
| --- | --- | --- | --- | --- | --- | --- | --- | --- | --- | --- |
| **Lineage** | **Organism** | **Nomenclature** | **SMART Domain** | **Start amino acid** | **End amino acid** | **E-Value** | **SMART Domain** | **Start amino acid** | **End amino acid** | **E-Value** |
| **Algae** | ***Chlamydomonas reinhardtii*** | ***Cr09g386750*** | **HATPase_c** | **30** | **185** | **7.94E-08** | **HSP90** | **187** | **699** | **5.60E-246** |
|  |  | ***Cr12g514850*** | **HATPase_c** | **103** | **259** | **7.29E-07** | **HSP90** | **261** | **787** | **1.50E-189** |
|  |  | ***Cr02g080650*** | **HATPase_c** | **92** | **249** | **1.27E-06** | **HSP90** | **251** | **857** | **9.60E-202** |
| **Mosses** | ***Physcomitrella patens*** | ***Pp3c156620V3*** | **HATPase_c** | **34** | **189** | **4.96E-09** | **HSP90** | **191** | **704** | **7.00E-244** |
|  |  | ***Pp3c156622V3*** | **HATPase_c** | **34** | **189** | **4.96E-09** | **HSP90** | **191** | **704** | **7.00E-244** |
|  |  | ***Pp3c1512510V3*** | **HATPase_c** | **34** | **189** | **2.41E-07** | **HSP90** | **191** | **702** | **8.30E-241** |
|  |  | ***Pp3c1512515V3*** | **HATPase_c** | **34** | **189** | **2.41E-07** | **HSP90** | **191** | **702** | **8.30E-241** |
|  |  | ***Pp3c96690V3*** | **HATPase_c** | **31** | **186** | **6.45E-08** | **HSP90** | **188** | **704** | **8.40E-244** |
|  |  | ***Pp3c96640V3*** | **HATPase_c** | **31** | **186** | **6.45E-08** | **HSP90** | **188** | **704** | **8.40E-244** |
|  |  | ***Pp3c154270V3*** | **HATPase_c** | **31** | **186** | **1.73E-08** | **HSP90** | **188** | **701** | **7.60E-244** |
|  |  | ***Pp3c143360V3*** | **HATPase_c** | **110** | **267** | **9.62E-07** | **HSP90** | **269** | **808** | **1.30E-220** |
|  |  | ***Pp3c4810V3*** | **HATPase_c** | **120** | **281** | **1.07E-04** | **HSP90** | **284** | **792** | **5.00E-193** |
|  |  | ***Pp3c1222440V3*** | **HATPase_c** | **127** | **288** | **9.48E-06** | **HSP90** | **291** | **800** | **1.10E-191** |
|  |  | ***Pp3c1915000V3*** | **HATPase_c** | **139** | **301** | **7.07E-05** | **HSP90** | **304** | **804** | **1.20E-180** |
| **Monocots** | ***Brachypodium distachyon*** | ***Bd5g02037*** | **HATPase_c** | **36** | **174** | **3.76E-09** | **HSP90** | **193** | **710** | **4.30E-243** |
|  |  | ***Bd3g39620*** | **HATPase_c** | **28** | **182** | **5.16E-07** | **HSP90** | **184** | **699** | **6.20E-242** |
|  |  | ***Bd3g39590*** | **HATPase_c** | **28** | **182** | **1.20E-07** | **HSP90** | **184** | **700** | **2.90E-242** |
|  |  | ***Bd3g39630*** | **HATPase_c** | **28** | **182** | **1.12E-07** | **HSP90** | **184** | **699** | **1.30E-241** |
|  |  | ***Bd1g30130*** | **HATPase_c** | **101** | **258** | **4.32E-09** | **HSP90** | **260** | **801** | **5.70E-222** |
|  |  | ***Bd4g06370*** | **HATPase_c** | **129** | **291** | **4.67E-05** | **HSP90** | **293** | **803** | **2.00E-190** |
|  |  | ***Bd4g32941*** | **HATPase_c** | **105** | **267** | **3.25E-04** | **HSP90** | **270** | **780** | **1.20E-195** |
|  |  | ***Bd3g38897*** | **HATPase_c** | **98** | **260** | **6.45E-08** | **HSP90** | **263** | **770** | **6.60E-196** |
|  | ***Oryza sativa*** | ***Os04g01740*** | **HATPase_c** | **35** | **173** | **4.96E-09** | **HSP90** | **192** | **703** | **1.10E-237** |
|  |  | ***Os08g39140*** | **HATPase_c** | **28** | **183** | **1.06E-08** | **HSP90** | **185** | **699** | **1.80E-243** |
|  |  | ***Os09g30412*** | **HATPase_c** | **28** | **183** | **2.62E-08** | **HSP90** | **175** | **699** | **5.60E-244** |
|  |  | ***Os09g30418*** | **HATPase_c** | **28** | **183** | **2.62E-08** | **HSP90** | **175** | **701** | **7.80E-242** |
|  |  | ***Os09g29840*** | **HATPase_c** | **105** | **267** | **6.80E-07** | **HSP90** | **270** | **779** | **5.90E-195** |
|  |  | ***Os08g38086*** | **HATPase_c** | **74** | **236** | **8.51E-08** | **HSP90** | **239** | **746** | **1.00E-193** |
|  |  | ***Os12g32986*** | **HATPase_c** | **130** | **292** | **1.13E-03** | **HSP90** | **294** | **800** | **5.50E-188** |
|  |  | ***Os06g50300*** | **HATPase_c** | **101** | **258** | **2.66E-09** | **HSP90** | **260** | **804** | **4.50E-221** |
|  | ***Triticum aestivum*** | ***Ta2DS3B16D8173*** | **HATPase_c** | **36** | **174** | **8.06E-09** | **HSP90** | **193** | **712** | **1.3E-241** |
|  |  | ***Ta2AS67EFE0FAE*** | **HATPase_c** | **153** | **291** | **8.06E-09** | **HSP90** | **310** | **818** | **5.9E-243** |
|  |  | ***Ta2BSF828BA5F41*** | **HATPase_c** | **37** | **175** | **3.76E-09** | **HSP90** | **194** | **712** | **5.7E-243** |
|  |  | ***Ta2BSF828BA5F4*** | **HATPase_c** | **37** | **175** | **3.76E-09** | **HSP90** | **194** | **712** | **5.7E-243** |
|  |  | ***Ta7BS1A6D16C6B*** | **HATPase_c** | **28** | **183** | **2.62E-08** | **HSP90** | **185** | **700** | **1.1E-228** |
|  |  | ***Ta7DSCB359539B*** | **HATPase_c** | **28** | **183** | **4.89E-08** | **HSP90** | **185** | **700** | **3E-228** |
|  |  | ***Ta5DL89CF7F5DE*** | **HATPase_c** | **28** | **183** | **9.77E-08** | **HSP90** | **185** | **700** | **5.4E-243** |
|  |  | ***Ta5BL0F3A986F9*** | **HATPase_c** | **28** | **183** | **9.77E-08** | **HSP90** | **185** | **700** | **5.4E-243** |
|  |  | ***Ta7AS76670DCAB*** | **HATPase_c** | **1** | **97** | **7.20E-07** | **HSP90** | **100** | **615** | **6.8E-229** |
|  |  | ***Ta5AL0C2D144B0*** | **HATPase_c** | **1** | **97** | **3.60E-06** | **HSP90** | **100** | **615** | **5.1E-241** |
|  |  | ***Ta5BL37ECD3B1E*** | **HATPase_c** | **11** | **173** | **1.25E-05** | **HSP90** | **176** | **688** | **2.9E-193** |
|  |  | ***Ta5DL5A546D5B3*** | **HATPase_c** | **11** | **173** | **1.25E-05** | **HSP90** | **176** | **687** | **2.2E-193** |
|  |  | ***Ta5AS5B7BFBD23*** | **HATPase_c** | **11** | **173** | **0.00184** | **HSP90** | **175** | **684** | **2.3E-185** |
|  |  | ***Ta7BL870E640C5*** | **HATPase_c** | **11** | **168** | **2.58E-07** | **HSP90** | **170** | **712** | **1.2E-218** |
|  |  | ***Ta5AL1DA3B4631*** | **HATPase_c** | **11** | **173** | **3.85E-06** | **HSP90** | **176** | **687** | **2.1E-193** |
|  |  | ***Ta5DSAC5D29D23*** | **HATPase_c** | **130** | **292** | **1.84E-03** | **HSP90** | **294** | **803** | **1.4E-179** |
|  |  | ***Ta7AL1A21E8798*** | **HATPase_c** | **11** | **168** | **2.58E-07** | **HSP90** | **170** | **553** | **1.1E-158** |
|  |  | ***Ta5BSAB86BB5DE*** | **HATPase_c** | **132** | **294** | **1.60E-03** | **HSP90** | **296** | **805** | **3.5E-179** |
|  | ***Zea mays*** | ***Zm5G833699*** | **HATPase_c** | **37** | **193** | **1.95E-07** | **HSP90** | **195** | **714** | **1.50E-244** |
|  |  | ***Zm2G069651*** | **HATPase_c** | **28** | **183** | **3.70E-08** | **HSP90** | **185** | **699** | **3.80E-243** |
|  |  | ***Zm2G112165*** | **HATPase_c** | **28** | **183** | **1.29E-07** | **HSP90** | **185** | **698** | **5.40E-244** |
|  |  | ***Zm2G012631*** | **HATPase_c** | **28** | **183** | **3.01E-08** | **HSP90** | **185** | **699** | **1.40E-242** |
|  |  | ***Zm2G141931*** | **HATPase_c** | **100** | **257** | **6.11E-09** | **HSP90** | **259** | **801** | **6.40E-222** |
|  |  | ***Zm2G399073*** | **HATPase_c** | **293** | **450** | **1.14E-08** | **HSP90** | **452** | **993** | **6.90E-218** |
|  |  | ***Zm2G024668*** | **HATPase_c** | **28** | **183** | **1.31E-08** | **HSP90** | **185** | **381** | **1.40E-70** |
|  |  | ***Zm2G002220*** | **HATPase_c** | **100** | **262** | **1.56E-06** | **HSP90** | **265** | **776** | **3.50E-197** |
|  |  | ***Zm5G813217*** | **HATPase_c** | **100** | **262** | **4.89E-08** | **HSP90** | **265** | **739** | **8.30E-193** |
| **Dicots** | ***Arabidopsis thaliana*** | ***At5G52640*** | **HATPase_c** | **32** | **187** | **8.31E-11** | **HSP90** | **189** | **705** | **4.10E-244** |
|  |  | ***At5G56000*** | **HATPase_c** | **27** | **182** | **7.52E-09** | **HSP90** | **184** | **699** | **2.60E-240** |
|  |  | ***At5G56010*** | **HATPase_c** | **27** | **182** | **6.54E-09** | **HSP90** | **184** | **699** | **4.70E-241** |
|  |  | ***At5G56030*** | **HATPase_c** | **46** | **211** | **2.07E-02** | **HSP90** | **213** | **728** | **3.40E-242** |
|  |  | ***At4G24190*** | **HATPase_c** | **99** | **256** | **4.56E-08** | **HSP90** | **258** | **802** | **1.10E-223** |
|  |  | ***At2G04030*** | **HATPase_c** | **99** | **261** | **2.54E-06** | **HSP90** | **264** | **770** | **1.40E-193** |
|  |  | ***At3G07770*** | **HATPase_c** | **117** | **279** | **6.52E-03** | **HSP90** | **281** | **793** | **4.90E-191** |
|  | ***Glycine max*** | ***Gm09G131500*** | **HATPase_c** | **27** | **182** | **1.35E-10** | **HSP90** | **184** | **699** | **8.70E-246** |
|  |  | ***Gm16G178800*** | **HATPase_c** | **27** | **182** | **2.35E-10** | **HSP90** | **184** | **699** | **7.40E-245** |
|  |  | ***Gm08G332900*** | **HATPase_c** | **27** | **182** | **3.05E-09** | **HSP90** | **184** | **699** | **3.40E-243** |
|  |  | ***Gm14G011600*** | **HATPase_c** | **28** | **183** | **1.14E-08** | **HSP90** | **185** | **700** | **3.20E-244** |
|  |  | ***Gm18G074100*** | **HATPase_c** | **27** | **182** | **1.61E-08** | **HSP90** | **184** | **700** | **2.80E-243** |
|  |  | ***Gm02G302500*** | **HATPase_c** | **28** | **183** | **1.14E-08** | **HSP90** | **185** | **700** | **4.50E-244** |
|  |  | ***Gm08G032900*** | **HATPase_c** | **43** | **140** | **7.90E-05** | **HSP90** | **143** | **623** | **3.70E-207** |
|  |  | ***Gm17G258700*** | **HATPase_c** | **100** | **257** | **2.48E-09** | **HSP90** | **259** | **805** | **1.40E-221** |
|  |  | ***Gm14G219700*** | **HATPase_c** | **100** | **257** | **2.85E-09** | **HSP90** | **259** | **748** | **1.80E-201** |
|  |  | ***Gm02G124500*** | **HATPase_c** | **104** | **266** | **8.84E-06** | **HSP90** | **269** | **779** | **7.60E-197** |
|  |  | ***Gm02G305600*** | **HATPase_c** | **110** | **272** | **1.49E-02** | **HSP90** | **274** | **787** | **5.40E-192** |
|  |  | ***Gm01G068000*** | **HATPase_c** | **103** | **265** | **6.50E-04** | **HSP90** | **268** | **778** | **2.70E-196** |
|  |  | ***Gm14G007700*** | **HATPase_c** | **117** | **279** | **1.58E-02** | **HSP90** | **281** | **793** | **2.10E-191** |
|  | ***Medicago truncatula*** | ***Mt6g452990*** | **HATPase_c** | **27** | **182** | **1.26E-10** | **HSP90** | **184** | **699** | **1.30E-242** |
|  |  | ***Mt1g099840*** | **HATPase_c** | **32** | **187** | **2.70E-10** | **HSP90** | **189** | **689** | **2.20E-227** |
|  |  | ***Mt5g096460*** | **HATPase_c** | **27** | **182** | **1.08E-09** | **HSP90** | **184** | **699** | **1.40E-242** |
|  |  | ***Mt5g096430*** | **HATPase_c** | **27** | **182** | **1.08E-09** | **HSP90** | **184** | **699** | **1.40E-242** |
|  |  | ***Mt5g097320*** | **HATPase_c** | **112** | **274** | **3.34E-02** | **HSP90** | **276** | **791** | **2.20E-192** |
|  |  | ***Mt1g025430*** | **HATPase_c** | **100** | **257** | **1.16E-09** | **HSP90** | **259** | **807** | **3.50E-221** |
|  | ***Gossypium raimondii*** | ***Gr004G138600*** | **HATPase_c** | **33** | **188** | **3.10E-10** | **HSP90** | **190** | **704** | **1.60E-243** |
|  |  | ***Gr008G274600*** | **HATPase_c** | **32** | **187** | **2.89E-10** | **HSP90** | **189** | **703** | **2.70E-243** |
|  |  | ***Gr003G155600*** | **HATPase_c** | **34** | **189** | **1.64E-09** | **HSP90** | **191** | **707** | **1.90E-243** |
|  |  | ***Gr002G103000*** | **HATPase_c** | **27** | **182** | **4.25E-08** | **HSP90** | **184** | **698** | **2.00E-240** |
|  |  | ***Gr004G033900*** | **HATPase_c** | **27** | **182** | **4.96E-09** | **HSP90** | **184** | **698** | **5.00E-240** |
|  |  | ***Gr013G150300*** | **HATPase_c** | **27** | **182** | **3.05E-09** | **HSP90** | **184** | **699** | **3.40E-241** |
|  |  | ***Gr004G034000*** | **HATPase_c** | **27** | **182** | **3.27E-09** | **HSP90** | **174** | **666** | **5.90E-224** |
|  |  | ***Gr002G122800*** | **HATPase_c** | **97** | **254** | **1.36E-06** | **HSP90** | **256** | **800** | **1.40E-216** |
|  |  | ***Gr001G220600*** | **HATPase_c** | **118** | **280** | **6.65E-03** | **HSP90** | **282** | **794** | **4.60E-193** |
|  |  | ***Gr013G098300*** | **HATPase_c** | **90** | **254** | **1.52E-02** | **HSP90** | **257** | **758** | **3.60E-176** |
|  |  | ***Gr005G148100*** | **HATPase_c** | **100** | **262** | **1.54E-05** | **HSP90** | **265** | **599** | **1.30E-133** |
|  |  | ***Gr010G003000*** | **HATPase_c** | **97** | **254** | **2.96E-07** | **HSP90** | **536** | **1083** | **4.00E-219** |
|  |  |  | **HATPase_c** | **377** | **534** | **2.96E-07** |  |  |  |  |
|  | | | | | | | | | | |

**B. The parameters of conserved Hsp90 domain from Pfam**

| **Lineage** | **Organism** | **Nnomenclature** | **Family** | **Description** | **Alignment** | | **Bit Score** | **E-Value** | **Family** | **Description** | **Alignment** | | **Bit Score** | **E-Value** |
| --- | --- | --- | --- | --- | --- | --- | --- | --- | --- | --- | --- | --- | --- | --- |
|  |  |  |  |  | **Start** | **End** |  |  |  |  | **Start** | **End** |  |  |
| **Algae** | | ***Cr09g386750*** | **PF02518** | **HATPase-c** | **30** | **185** | **57.9** | **1.2E-15** | **PF00183** | **HSP90** | **187** | **695** | **802.6** | **1.5E-241** |
|  |  | ***Cr12g514850*** | **PF02518** | **HATPase-c** | **103** | **259** | **49.6** | **4.5E-13** | **PF00183** | **HSP90** | **261** | **784** | **620.3** | **2.6E-186** |
|  |  | ***Cr02g080650*** | **PF02518** | **HATPase-c** | **92** | **249** | **46.7** | **3.5E-12** | **PF00183** | **HSP90** | **251** | **805** | **663.1** | **2.9E-199** |
| **Mosses** | ***Physcomitrella patens*** | ***Pp3c156620V3*** | **PF02518** | **HATPase-c** | **34** | **189** | **56.6** | **2.9E-15** | **PF00183** | **HSP90** | **191** | **702** | **796.9** | **8.3E-240** |
|  |  | ***Pp3c156622V3*** | **PF02518** | **HATPase-c** | **34** | **189** | **56.6** | **2.9E-15** | **PF00183** | **HSP90** | **191** | **702** | **796.9** | **8.3E-240** |
|  |  | ***Pp3c1512510V3*** | **PF02518** | **HATPase-c** | **34** | **189** | **55.1** | **8.9E-15** | **PF00183** | **HSP90** | **191** | **700** | **793.7** | **7.4E-239** |
|  |  | ***Pp3c1512515V3*** | **PF02518** | **HATPase-c** | **34** | **189** | **55.1** | **8.9E-15** | **PF00183** | **HSP90** | **191** | **700** | **793.7** | **7.4E-239** |
|  |  | ***Pp3c96690V3*** | **PF02518** | **HATPase-c** | **31** | **186** | **56.6** | **3.0E-15** | **PF00183** | **HSP90** | **188** | **702** | **796.4** | **1.2E-239** |
|  |  | ***Pp3c96640V3*** | **PF02518** | **HATPase-c** | **34** | **189** | **56.6** | **3.0E-15** | **PF00183** | **HSP90** | **191** | **705** | **796.4** | **1.2E-239** |
|  |  | ***Pp3c154270V3*** | **PF02518** | **HATPase-c** | **31** | **186** | **57** | **2.2E-15** | **PF00183** | **HSP90** | **188** | **699** | **796.7** | **9.3E-240** |
|  |  | ***Pp3c143360V3*** | **PF02518** | **HATPase-c** | **110** | **267** | **51.3** | **1.3E-13** | **PF00183** | **HSP90** | **269** | **805** | **722.7** | **2.4E-217** |
|  |  | ***Pp3c4810V3*** | **PF02518** | **HATPase-c** | **120** | **281** | **50.2** | **2.9E-13** | **PF00183** | **HSP90** | **284** | **788** | **641** | **1.4E-192** |
|  |  | ***Pp3c1222440V3*** | **PF02518** | **HATPase-c** | **127** | **288** | **50.7** | **2.0E-13** | **PF00183** | **HSP90** | **291** | **796** | **636.5** | **3.2E-191** |
|  |  | ***Pp3c1915000V3*** | **PF02518** | **HATPase-c** | **139** | **301** | **47.4** | **2.2E-12** | **PF00183** | **HSP90** | **304** | **802** | **600.3** | **3E-180** |
| **Monocots** | ***Brachypodium distachyon*** | ***Bd5g02037*** | **PF02518** | **HATPase-c** | **36** | **191** | **54.6** | **1.30E-14** | **PF00183** | **HSP90** | **193** | **708** | **792.4** | **1.9E-238** |
|  |  | ***Bd3g39620*** | **PF02518** | **HATPase-c** | **28** | **182** | **54.8** | **1.1E-14** | **PF00183** | **HSP90** | **184** | **697** | **788.8** | **3.0E-237** |
|  |  | ***Bd3g39590*** | **PF02518** | **HATPase-c** | **28** | **182** | **56.6** | **3.0E-15** | **PF00183** | **HSP90** | **184** | **698** | **789.9** | **1.1E-237** |
|  |  | ***Bd3g39630*** | **PF02518** | **HATPase-c** | **28** | **182** | **56.0** | **4.7E-15** | **PF00183** | **HSP90** | **184** | **697** | **787.9** | **4.2E-237** |
|  |  | ***Bd1g30130*** | **PF02518** | **HATPase-c** | **101** | **258** | **52.2** | **6.8E-14** | **PF00183** | **HSP90** | **260** | **796** | **727.0** | **1.3E-218** |
|  |  | ***Bd4g06370*** | **PF02518** | **HATPase-c** | **129** | **191** | **47.7** | **1.8E-12** | **PF00183** | **HSP90** | **293** | **799** | **624.4** | **1.5E-187** |
|  |  | ***Bd4g32941*** | **PF02518** | **HATPase-c** | **105** | **267** | **49.2** | **6.0E-13** | **PF00183** | **HSP90** | **270** | **777** | **641.1** | **1.3E-192** |
|  |  | ***Bd3g38897*** | **PF02518** | **HATPase-c** | **98** | **260** | **53.6** | **2.5E-14** | **PF00183** | **HSP90** | **263** | **767** | **642.2** | **6.1E-193** |
|  | ***Oryza sativa*** | ***Os04g01740*** | **PF02518** | **HATPase-c** | **35** | **190** | **51.4** | **1.2E-13** | **PF00183** | **HSP90** | **192** | **701** | **775.0** | **3.4E-233** |
|  |  | ***Os08g39140*** | **PF02518** | **HATPase-c** | **28** | **183** | **58.0** | **1.1E-15** | **PF00183** | **HSP90** | **185** | **697** | **796.7** | **9.3E-240** |
|  |  | ***Os09g30412*** | **PF02518** | **HATPase-c** | **28** | **183** | **56.1** | **4.1E-15** | **PF00183** | **HSP90** | **185** | **697** | **798.5** | **2.7E-240** |
|  |  | ***Os09g30418*** | **PF02518** | **HATPase-c** | **28** | **183** | **55.7** | **5.5E-15** | **PF00183** | **HSP90** | **185** | **697** | **797.5** | **5.4E-240** |
|  |  | ***Os09g29840*** | **PF02518** | **HATPase-c** | **105** | **267** | **54.0** | **1.9E-14** | **PF00183** | **HSP90** | **270** | **775** | **646.4** | **3.3E-194** |
|  |  | ***Os08g38086*** | **PF02518** | **HATPase-c** | **74** | **236** | **52.9** | **4.2E-14** | **PF00183** | **HSP90** | **239** | **743** | **643.0** | **3.5E-193** |
|  |  | ***Os12g32986*** | **PF02518** | **HATPase-c** | **130292** | **1** | **265** | **3.4E-12** | **PF00183** | **HSP90** | **294** | **798** | **616.2** | **4.6E-185** |
|  |  | ***Os06g50300*** | **PF02518** | **HATPase-c** | **101** | **258** | **54.5** | **1.3E-14** | **PF00183** | **HSP90** | **260** | **800** | **724.1** | **9.5E-218** |
|  | ***Triticum aestivum*** | ***Ta2DS3B16D8173*** | **PF02518** | **HATPase-c** | **36** | **193** | **56.4** | **3.5E-15** | **PF00183** | **HSP90** | **193** | **710** | **795.8** | **1.7E-239** |
|  |  | ***Ta2AS67EFE0FAE*** | **PF02518** | **HATPase-c** | **153** | **308** | **56.0** | **4.5E-15** | **PF00183** | **HSP90** | **310** | **826** | **800.3** | **7.7E-241** |
|  |  | ***Ta2BSF828BA5F41*** | **PF02518** | **HATPase-c** | **37** | **192** | **55.3** | **7.7E-15** | **PF00183** | **HSP90** | **194** | **7101** | **793.1** | **1.2E-238** |
|  |  | ***Ta2BSF828BA5F4*** | **PF02518** | **HATPase-c** | **37** | **192** | **55.3** | **7.7E-15** | **PF00183** | **HSP90** | **194** | **710** | **793.1** | **1.2E-238** |
|  |  | ***Ta7BS1A6D16C6B*** | **PF02518** | **HATPase-c** | **28** | **183** | **59.4** | **4.0E-16** | **PF00183** | **HSP90** | **185** | **698** | **753.8** | **9.4E-227** |
|  |  | ***Ta7DSCB359539B*** | **PF02518** | **HATPase-c** | **28** | **183** | **58.3** | **8.9E-16** | **PF00183** | **HSP90** | **185** | **698** | **752.3** | **2.7E-226** |
|  |  | ***Ta5DL89CF7F5DE*** | **PF02518** | **HATPase-c** | **28** | **183** | **55.6** | **6.2E-15** | **PF00183** | **HSP90** | **185** | **698** | **793.6** | **8.2E-239** |
|  |  | ***Ta5BL0F3A986F9*** | **PF02518** | **HATPase-c** | **28** | **183** | **55.6** | **6.2E-15** | **PF00183** | **HSP90** | **185** | **698** | **793.6** | **8.2E-239** |
|  |  | ***Ta7AS76670DCAB*** | **PF02518** | **HATPase-c** | **1** | **98** | **17.1** | **0.0057** | **PF00183** | **HSP90** | **100** | **613** | **754.4** | **6.1E-227** |
|  |  | ***Ta5AL0C2D144B0*** | **PF02518** | **HATPase-c** |  |  |  |  | **PF00183** | **HSP90** | **100** | **613** | **794.2** | **5.3E-239** |
|  |  | ***Ta5BL37ECD3B1E*** | **PF02518** | **HATPase-c** | **11** | **173** | **52.8** | **4.4E-14** | **PF00183** | **HSP90** | **176** | **681** | **642.5** | **4.9E-193** |
|  |  | ***Ta5DL5A546D5B3*** | **PF02518** | **HATPase-c** | **11** | **173** | **52.8** | **4.4E-14** | **PF00183** | **HSP90** | **176** | **681** | **642.3** | **5.7E-193** |
|  |  | ***Ta5AS5B7BFBD23*** | **PF02518** | **HATPase-c** | **11** | **173** | **47.8** | **1.7E-12** | **PF00183** | **HSP90** | **175** | **681** | **615.6** | **7.0E-185** |
|  |  | ***Ta7BL870E640C5*** | **PF02518** | **HATPase-c** | **11** | **168** | **52.8** | **4.6E-14** | **PF00183** | **HSP90** | **170** | **707** | **724.9** | **5.3E-218** |
|  |  | ***Ta5AL1DA3B4631*** | **PF02518** | **HATPase-c** | **11** | **173** | **53.5** | **2.8E-14** | **PF00183** | **HSP90** | **176** | **679** | **642.3** | **5.6E-193** |
|  |  | ***Ta5DSAC5D29D23*** | **PF02518** | **HATPase-c** | **130** | **292** | **47.8** | **1.6E-12** | **PF00183** | **HSP90** | **294** | **800** | **596.6** | **4.1E-179** |
|  |  | ***Ta7AL1A21E8798*** | **PF02518** | **HATPase-c** | **11** | **168** | **53.4** | **2.9E-14** | **PF00183** | **HSP90** | **170** | **553** | **527.9** | **2.8E-158** |
|  |  | ***Ta5BSAB86BB5DE*** | **PF02518** | **HATPase-c** | **132** | **294** | **47.8** | **1.60E-12** | **PF00183** | **HSP90** | **296** | **802** | **595.2** | **1.10E-178** |
|  | ***Zea mays*** | ***Zm5G833699*** | **PF02518** | **HATPase-c** | **37** | **193** | **56.5** | **3.1E-15** | **PF00183** | **HSP90** | **195** | **712** | **797.2** | **6.5E-240** |
|  |  | ***Zm2G069651*** | **PF02518** | **HATPase-c** | **28** | **183** | **57.8** | **1.2E-15** | **PF00183** | **HSP90** | **185** | **697** | **801.3** | **3.8E-241** |
|  |  | ***Zm2G112165*** | **PF02518** | **HATPase-c** | **28** | **183** | **56.9** | **2.4E-15** | **PF00183** | **HSP90** | **185** | **696** | **804.0** | **5.8E-242** |
|  |  | ***Zm2G012631*** | **PF02518** | **HATPase-c** | **28** | **183** | **58.2** | **9.4E-16** | **PF00183** | **HSP90** | **185** | **697** | **790.9** | **5.3E-238** |
|  |  | ***Zm2G141931*** | **PF02518** | **HATPase-c** | **100** | **257** | **54.4** | **1.4E-14** | **PF00183** | **HSP90** | **259** | **796** | **728.1** | **5.9E-219** |
|  |  | ***Zm2G399073*** | **PF02518** | **HATPase-c** | **293** | **450** | **53.9** | **2.1E-14** | **PF00183** | **HSP90** | **452** | **989** | **721.5** | **5.8E-217** |
|  |  | ***Zm2G024668*** | **PF02518** | **HATPase-c** | **28** | **183** | **60.4** | **2.0E-16** | **PF00183** | **HSP90** | **185** | **351** | **236.4** | **6.0E-70** |
|  |  | ***Zm2G002220*** | **PF02518** | **HATPase-c** | **100** | **262** | **48.7** | **8.6E-13** | **PF00183** | **HSP90** | **265** | **773** | **646.4** | **3.3E-194** |
|  |  | ***Zm5G813217*** | **PF02518** | **HATPase-c** | **100** | **262** | **50.4** | **2.4E-13** | **PF00183** | **HSP90** | **265** | **739** | **640.4** | **2.1E-192** |
| **Dicots** | ***Arabidopsis thaliana*** | ***At5G52640*** | **PF02518** | **HATPase-c** | **32** | **187** | **58.4** | **8.1E-16** | **PF00183** | **HSP90** | **189** | **703** | **804** | **5.6E-242** |
|  |  | ***At5G56000*** | **PF02518** | **HATPase-c** | **27** | **182** | **58.1** | **1.0E-15** | **PF00183** | **HSP90** | **184** | **696** | **786.5** | **1.2E-236** |
|  |  | ***At5G56010*** | **PF02518** | **HATPase-c** | **27** | **182** | **57.9** | **1.2E-15** | **PF00183** | **HSP90** | **184** | **696** | **788.8** | **2.4E-237** |
|  |  | ***At5G56030*** | **PF02518** | **HATPase-c** | **47** | **211** | **43.3** | **4.1E-11** | **PF00183** | **HSP90** | **213** | **725** | **791.0** | **5.0E-238** |
|  |  | ***At4G24190*** | **PF02518** | **HATPase-c** | **99** | **256** | **52.9** | **4.3E-14** | **PF00183** | **HSP90** | **258** | **798** | **730.5** | **1.0E-219** |
|  |  | ***At2G04030*** | **PF02518** | **HATPase-c** | **99** | **261** | **53.4** | **3.0E-14** | **PF00183** | **HSP90** | **264** | **767** | **633.6** | **2.5E-190** |
|  |  | ***At3G07770*** | **PF02518** | **HATPase-c** | **117** | **279** | **51.3** | **1.3E-13** | **PF00183** | **HSP90** | **281** | **789** | **626.6** | **3.2E-188** |
|  | ***Glycine max*** | ***Gm09G131500*** | **PF02518** | **HATPase-c** | **27** | **182** | **62.9** | **3.2E-17** | **PF00183** | **HSP90** | **184** | **697** | **801.4** | **3.5E-241** |
|  |  | ***Gm16G178800*** | **PF02518** | **HATPase-c** | **27** | **182** | **60.6** | **1.7E-16** | **PF00183** | **HSP90** | **184** | **697** | **798.3** | **3.0E-240** |
|  |  | ***Gm08G332900*** | **PF02518** | **HATPase-c** | **27** | **182** | **61.5** | **8.7E-17** | **PF00183** | **HSP90** | **184** | **696** | **793.2** | **1.1E-238** |
|  |  | ***Gm14G011600*** | **PF02518** | **HATPase-c** | **28** | **183** | **62.3** | **5.0E-17** | **PF00183** | **HSP90** | **185** | **697** | **796.5** | **1.1E-239** |
|  |  | ***Gm18G074100*** | **PF02518** | **HATPase-c** | **27** | **182** | **60.9** | **1.4E-16** | **PF00183** | **HSP90** | **184** | **696** | **794.4** | **4.5E-239** |
|  |  | ***Gm02G302500*** | **PF02518** | **HATPase-c** | **28** | **183** | **62.3** | **5.0E-17** | **PF00183** | **HSP90** | **185** | **696** | **796.6** | **7.9E-240** |
|  |  | ***Gm08G032900*** | **PF02518** | **HATPase-c** | **28** | **183** | **62.8** | **5.0E-19** | **PF00183** | **HSP90** | **143** | **623** | **683.5** | **1.9E-205** |
|  |  | ***Gm17G258700*** | **PF02518** | **HATPase-c** | **100** | **257** | **54.4** | **1.4E-14** | **PF00183** | **HSP90** | **259** | **748** | **725.2** | **4.5E-218** |
|  |  | ***Gm14G219700*** | **PF02518** | **HATPase-c** | **100** | **257** | **54.4** | **1.5E-14** | **PF00183** | **HSP90** | **259** | **748** | **660.4** | **1.9E-198** |
|  |  | ***Gm02G124500*** | **PF02518** | **HATPase-c** | **104** | **266** | **53.3** | **3.3E-14** | **PF00183** | **HSP90** | **269** | **777** | **644.4** | **1.4E-193** |
|  |  | ***Gm02G305600*** | **PF02518** | **HATPase-c** | **110** | **272** | **49.2** | **6.0E-13** | **PF00183** | **HSP90** | **274** | **784** | **629.2** | **5.4E-189** |
|  |  | ***Gm01G068000*** | **PF02518** | **HATPase-c** | **103** | **265** | **50.8** | **1.9E-13** | **PF00183** | **HSP90** | **268** | **776** | **642.5** | **5.0E-193** |
|  |  | ***Gm14G007700*** | **PF02518** | **HATPase-c** | **117** | **279** | **49.2** | **5.8E-13** | **PF00183** | **HSP90** | **281** | **790** | **635.5** | **6.5E-191** |
|  | ***Medicago truncatula*** | ***Mt6g452990*** | **PF02518** | **HATPase-c** | **27** | **182** | **60.3** | **2.10E-16** | **PF00183** | **HSP90** | **184** | **697** | **799.3** | **1.50E-240** |
|  |  | ***Mt1g099840*** | **PF02518** | **HATPase-c** | **32** | **187** | **61.2** | **1.1E-16** | **PF00183** | **HSP90** | **189** | **687** | **741.3** | **5.9E-223** |
|  |  | ***Mt5g096460*** | **PF02518** | **HATPase-c** | **27** | **182** | **61.5** | **9.0E-17** | **PF00183** | **HSP90** | **184** | **696** | **791.4** | **3.6E-238** |
|  |  | ***Mt5g096430*** | **PF02518** | **HATPase-c** | **27** | **182** | **61.5** | **9.0E-17** | **PF00183** | **HSP90** | **184** | **696** | **791.4** | **3.6E-238** |
|  |  | ***Mt5g097320*** | **PF02518** | **HATPase-c** | **112** | **274** | **49.7** | **4.0E-13** | **PF00183** | **HSP90** | **276** | **787** | **630.2** | **2.8E-189** |
|  |  | ***Mt1g025430*** | **PF02518** | **HATPase-c** | **100** | **257** | **54.0** | **1.9E-14** | **PF00183** | **HSP90** | **259** | **802** | **725.4** | **3.7E-218** |
|  | ***Gossypium raimondii*** | ***Gr004G138600*** | **PF02518** | **HATPase-c** | **33** | **188** | **59.9** | **2.7E-16** | **PF00183** | **HSP90** | **190** | **702** | **802.3** | **1.9E-241** |
|  |  | ***Gr008G274600*** | **PF02518** | **HATPase-c** | **32** | **187** | **61.1** | **1.2E-16** | **PF00183** | **HSP90** | **189** | **701** | **801.5** | **3.3E-241** |
|  |  | ***Gr003G155600*** | **PF02518** | **HATPase-c** | **34** | **189** | **58.1** | **1.0E-15** | **PF00183** | **HSP90** | **191** | **705** | **802.0** | **2.2E-241** |
|  |  | ***Gr002G103000*** | **PF02518** | **HATPase-c** | **27** | **182** | **57.0** | **2.3E-15** | **PF00183** | **HSP90** | **184** | **696** | **792.8** | **1.4E-238** |
|  |  | ***Gr004G033900*** | **PF02518** | **HATPase-c** | **27** | **182** | **62** | **6.3E-17** | **PF00183** | **HSP90** | **184** | **696** | **791.2** | **4.4E-238** |
|  |  | ***Gr013G150300*** | **PF02518** | **HATPase-c** | **27** | **182** | **61.1** | **1.2E-16** | **PF00183** | **HSP90** | **184** | **696** | **795.0** | **3.0E-239** |
|  |  | ***Gr004G034000*** | **PF02518** | **HATPase-c** | **27** | **182** | **62.2** | **5.3E-17** | **PF00183** | **HSP90** | **174** | **663** | **738.5** | **4.0E-222** |
|  |  | ***Gr002G122800*** | **PF02518** | **HATPase-c** | **97** | **254** | **52.3** | **6.5E-14** | **PF00183** | **HSP90** | **256** | **796** | **717.0** | **1.3E-215** |
|  |  | ***Gr001G220600*** | **PF02518** | **HATPase-c** | **118** | **280** | **49.5** | **4.9E-13** | **PF00183** | **HSP90** | **282** | **790** | **640.4** | **2.2E-192** |
|  |  | ***Gr013G098300*** | **PF02518** | **HATPase-c** | **90** | **254** | **44.5** | **1.8E-11** | **PF00183** | **HSP90** | **257** | **755** | **586.6** | **9.0E-176** |
|  |  | ***Gr005G148100*** | **PF02518** | **HATPase-c** | **100** | **262** | **51.6** | **1.0E-13** | **PF00183** | **HSP90** | **265** | **599** | **445.9** | **2.0E-133** |
|  |  | ***Gr010G003000*** | **PF02518** | **HATPase-c** | **97** | **254** | **49.9** | **3.6E-13** | **PF00183** | **HSP90** | **536** | **1080** | **727.0** | **1.2E-218** |
