# Supplementary material for: Genome-wide analysis of the Brachypodium distachyon (L.) P. Beauv. Hsp90 gene family reveals molecular evolution and expression profiling under drought and salt stresses
Source: PLoS One. 2017 Dec 7;12(12):e0189187. doi: 10.1371/journal.pone.0189187 (PMC5720741; doi:10.1371/journal.pone.0189187)
Supplement: S4 Table — (DOCX) [file pone.0189187.s007.docx]

| **S4 Table. Segmentally duplicates and tandem duplicates of Hsp90 genes in ten species** | | | | | |
| --- | --- | --- | --- | --- | --- |
| **Lineage** | **Organism** | **Segmentally duplicates** | | **Tandem duplicates** | |
| Algae | *Chlamydomonas reinhardtii* | 0 |  | 0 |  |
| Mosses | *Physcomitrella patens* | 0 |  | 2 | *Pp3c156620V3 & Pp3c156622V3* |
|  |  |  |  |  | *Pp3c1512510V3 & Pp3c1512515V3* |
| *Monocots* | *Brachypodium distachyon* | 1 | *Bd3g38897 & Bd4g32941* | 1 | *Bd3g39620 & Bd3g39630* |
|  | *Oryza sativa* | *2* | *Os08g39140 & Os09g30412* | *1* | *Os09g30412 & Os09g30418* |
|  |  |  | *Os08g38086 & Os09g29840* |  |  |
|  | *Zea mays* | 0 |  | 0 |  |
|  |  |  |  |  |  |
|  | *Triticum aestivum* | *0* |  | *0* |  |
| *Dicots* | *Arabidopsis thaliana* | 0 |  | 2 | At5G56000 & At5G56010 |
|  |  |  |  |  | *At5G56010& At5G56030* |
|  |  |  |  |  |  |
|  | *Glycine max* | 9 | *Gm02G302500 & Gm08G332900* | 0 |  |
|  |  |  | *Gm08G332900 & Gm14G011600* |  |  |
|  |  |  | *Gm08G332900 & Gm18G074100* |  |  |
|  |  |  | *Gm02G302500 & Gm14G011600* |  |  |
|  |  |  | *Gm14G011600 & Gm18G074100* |  |  |
|  |  |  | *Gm02G302500 & Gm18G074100* |  |  |
|  |  |  | *Gm14G219700 & Gm17G258700* |  |  |
|  |  |  | *Gm01G068000 & Gm02G124500* |  |  |
|  |  |  | *Gm02G305600 & Gm14G007700* |  |  |
|  | *Medicago truncatula* | 0 |  | 1 | *Mt5g096430 & Mt5g096460* |
|  |  |  |  |  |  |
|  |  |  |  |  |  |
|  | *Gossypium raimondii* | 3 | *Gr003G155600 & Gr004G138600* | 0 |  |
|  |  |  | *Gr002G103000 & Gr013G150300* |  |  |
|  |  |  | *Gr002G122800 & Gr010G003000* |  |  |
