# Supplementary material for: Genome-wide analysis of the Brachypodium distachyon (L.) P. Beauv. Hsp90 gene family reveals molecular evolution and expression profiling under drought and salt stresses
Source: PLoS One. 2017 Dec 7;12(12):e0189187. doi: 10.1371/journal.pone.0189187 (PMC5720741; doi:10.1371/journal.pone.0189187)
Supplement: S5 Table — (DOCX) [file pone.0189187.s008.docx]

**S5 Table. Subcellular localization of Hsp90 genes in ten species by three predicted tools**

| **TargetP1.1 prediction results** | | | | | | |
| --- | --- | --- | --- | --- | --- | --- |
| Name | Len | cTP | mTP | SP | other score | Location |
| At5G52640 | 705 | 0.123 | 0.053 | 0.289 | 0.609 | _ |
| At5G56000 | 699 | 0.082 | 0.062 | 0.483 | 0.449 | S |
| At5G56010 | 699 | 0.082 | 0.062 | 0.483 | 0.449 | S |
| At5G56030 | 728 | 0.156 | 0.063 | 0.351 | 0.307 | S |
| At4G24190 | 823 | 0.009 | 0.172 | 0.944 | 0.006 | S |
| At2G04030 | 780 | 0.92 | 0.335 | 0.013 | 0.013 | C |
| At3G07770 | 799 | 0.596 | 0.873 | 0.003 | 0.007 | M |
|  |  |  |  |  |  |  |
| Bd5g02037 | 710 | 0.108 | 0.083 | 0.192 | 0.712 | _ |
| Bd3g39620 | 699 | 0.101 | 0.061 | 0.323 | 0.492 | _ |
| Bd3g39590 | 700 | 0.102 | 0.061 | 0.335 | 0.495 | _ |
| Bd3g39630 | 699 | 0.101 | 0.061 | 0.323 | 0.492 | _ |
| Bd1g30130 | 807 | 0.044 | 0.081 | 0.925 | 0.007 | S |
| Bd4g06370 | 813 | 0.174 | 0.616 | 0.004 | 0.009 | M |
| Bd4g32941 | 794 | 0.795 | 0.707 | 0.006 | 0.009 | C |
| Bd3g38897 | 783 | 0.857 | 0.35 | 0.012 | 0.013 | C |
|  |  |  |  |  |  |  |
| Cr09g386750 | 705 | 0.153 | 0.052 | 0.331 | 0.561 | _ |
| Cr12g514850 | 810 | 0.741 | 0.642 | 0.002 | 0.005 | C |
| Cr02g080650 | 819 | 0.009 | 0.056 | 0.973 | 0.027 | S |
|  |  |  |  |  |  |  |
| Gm09G131500 | 699 | 0.071 | 0.073 | 0.48 | 0.458 | S |
| Gm16G178800 | 699 | 0.069 | 0.072 | 0.429 | 0.466 | _ |
| Gm08G332900 | 699 | 0.069 | 0.081 | 0.419 | 0.486 | _ |
| Gm14G011600 | 700 | 0.092 | 0.077 | 0.321 | 0.523 | _ |
| Gm18G074100 | 702 | 0.067 | 0.081 | 0.39 | 0.5 | _ |
| Gm02G302500 | 702 | 0.092 | 0.077 | 0.321 | 0.523 | _ |
| Gm08G032900 | 655 | 0.096 | 0.87 | 0.014 | 0.018 | M |
| Gm17G258700 | 814 | 0.007 | 0.089 | 0.973 | 0.011 | S |
| Gm14G219700 | 847 | 0.007 | 0.091 | 0.975 | 0.012 | S |
| Gm02G124500 | 794 | 0.867 | 0.366 | 0.01 | 0.01 | C |
| Gm02G305600 | 791 | 0.136 | 0.858 | 0.012 | 0.027 | M |
| Gm01G068000 | 793 | 0.725 | 0.243 | 0.047 | 0.008 | C |
| Gm14G007700 | 797 | 0.081 | 0.888 | 0.01 | 0.065 | M |
|  |  |  |  |  |  |  |
| Gr004G138600 | 704 | 0.104 | 0.089 | 0.168 | 0.763 | _ |
| Gr008G274600 | 703 | 0.065 | 0.044 | 0.476 | 0.585 | _ |
| Gr003G155600 | 707 | 0.1 | 0.093 | 0.171 | 0.762 | _ |
| Gr002G103000 | 699 | 0.078 | 0.063 | 0.406 | 0.497 | _ |
| Gr004G033900 | 699 | 0.066 | 0.082 | 0.387 | 0.52 | _ |
| Gr013G150300 | 699 | 0.066 | 0.081 | 0.384 | 0.521 | _ |
| Gr004G034000 | 666 | 0.068 | 0.079 | 0.41 | 0.516 | _ |
| Gr002G122800 | 809 | 0.051 | 0.052 | 0.877 | 0.042 | S |
| Gr001G220600 | 797 | 0.108 | 0.876 | 0.023 | 0.012 | M |
| Gr013G098300 | 777 | 0.006 | 0.052 | 0.922 | 0.144 | S |
| Gr005G148100 | 832 | 0.859 | 0.177 | 0.025 | 0.017 | C |
| Gr010G003000 | 1084 | 0.025 | 0.096 | 0.9 | 0.021 | S |
|  |  |  |  |  |  |  |
| Mt6g452990 | 699 | 0.077 | 0.06 | 0.433 | 0.453 | _ |
| Mt1g099840 | 689 | 0.108 | 0.069 | 0.185 | 0.764 | _ |
| Mt5g096460 | 699 | 0.062 | 0.082 | 0.456 | 0.49 | _ |
| Mt5g096430 | 699 | 0.062 | 0.082 | 0.456 | 0.49 | _ |
| Mt5g097320 | 792 | 0.115 | 0.817 | 0.033 | 0.032 | M |
| Mt1g025430 | 818 | 0.006 | 0.05 | 0.97 | 0.023 | S |
|  |  |  |  |  |  |  |
| Os04g01740 | 703 | 0.073 | 0.078 | 0.224 | 0.649 | _ |
| Os08g39140 | 699 | 0.1 | 0.059 | 0.289 | 0.515 | _ |
| Os09g30412 | 699 | 0.1 | 0.055 | 0.291 | 0.526 | _ |
| Os09g30418 | 830 | 0.1 | 0.055 | 0.291 | 0.526 | _ |
| Os09g29840 | 791 | 0.84 | 0.517 | 0.011 | 0.008 | C |
| Os08g38086 | 761 | 0.695 | 0.673 | 0.01 | 0.009 | C |
| Os12g32986 | 811 | 0.365 | 0.647 | 0.016 | 0.002 | M |
| Os06g50300 | 812 | 0.02 | 0.074 | 0.956 | 0.014 | S |
|  |  |  |  |  |  |  |
| Pp3c156620V3 | 704 | 0.114 | 0.084 | 0.191 | 0.752 | _ |
| Pp3c156622V3 | 704 | 0.114 | 0.084 | 0.191 | 0.752 | _ |
| Pp3c1512510V3 | 702 | 0.11 | 0.058 | 0.359 | 0.542 | _ |
| Pp3c1512515V3 | 702 | 0.11 | 0.058 | 0.359 | 0.542 | _ |
| Pp3c96690V3 | 704 | 0.085 | 0.084 | 0.222 | 0.717 | _ |
| Pp3c96640V3 | 707 | 0.131 | 0.068 | 0.313 | 0.616 | _ |
| Pp3c154270V3 | 701 | 0.086 | 0.084 | 0.21 | 0.718 | _ |
| Pp3c143360V3 | 849 | 0.001 | 0.583 | 0.891 | 0.007 | S |
| Pp3c4810V3 | 804 | 0.932 | 0.172 | 0.001 | 0.016 | C |
| Pp3c1222440V3 | 811 | 0.974 | 0.024 | 0.006 | 0.009 | C |
| Pp3c1915000V3 | 806 | 0.057 | 0.924 | 0.01 | 0.029 | M |
|  |  |  |  |  |  |  |
| Ta2DS3B16D8173 | 712 | 0.092 | 0.08 | 0.188 | 0.744 | _ |
| Ta2AS67EFE0FAE | 828 | 0.09 | 0.53 | 0 | 0.496 | M |
| Ta2BSF828BA5F41 | 712 | 0.066 | 0.072 | 0.272 | 0.68 | _ |
| Ta2BSF828BA5F4 | 712 | 0.066 | 0.072 | 0.272 | 0.68 | _ |
| Ta7BS1A6D16C6B | 700 | 0.088 | 0.07 | 0.326 | 0.517 | _ |
| Ta7DSCB359539B | 700 | 0.086 | 0.075 | 0.339 | 0.515 | _ |
| Ta5DL89CF7F5DE | 700 | 0.088 | 0.069 | 0.318 | 0.53 | _ |
| Ta5BL0F3A986F9 | 700 | 0.088 | 0.069 | 0.318 | 0.53 | _ |
| Ta7AS76670DCAB | 615 | 0.082 | 0.186 | 0.08 | 0.876 | _ |
| Ta5AL0C2D144B0 | 615 | 0.06 | 0.207 | 0.111 | 0.856 | _ |
| Ta5BL37ECD3B1E | 695 | 0.054 | 0.101 | 0.454 | 0.641 | _ |
| Ta5DL5A546D5B3 | 695 | 0.054 | 0.101 | 0.454 | 0.641 | _ |
| Ta5AS5B7BFBD23 | 694 | 0.044 | 0.102 | 0.39 | 0.666 | _ |
| Ta7BL870E640C5 | 716 | 0.065 | 0.072 | 0.466 | 0.699 | _ |
| Ta5AL1DA3B4631 | 695 | 0.051 | 0.104 | 0.453 | 0.64 | _ |
| Ta5DSAC5D29D23 | 813 | 0.222 | 0.667 | 0.004 | 0.006 | M |
| Ta7AL1A21E8798 | 553 | 0.065 | 0.072 | 0.466 | 0.699 | _ |
| Ta5BSAB86BB5DE | 815 | 0.149 | 0.732 | 0.005 | 0.006 | M |
|  |  |  |  |  |  |  |
| Zm5G833699 | 714 | 0.077 | 0.074 | 0.198 | 0.772 | _ |
| Zm2G069651 | 699 | 0.085 | 0.066 | 0.269 | 0.554 | _ |
| Zm2G112165 | 698 | 0.104 | 0.06 | 0.306 | 0.487 | _ |
| Zm2G012631 | 699 | 0.101 | 0.064 | 0.347 | 0.5 | _ |
| Zm2G141931 | 804 | 0.063 | 0.078 | 0.849 | 0.019 | S |
| Zm2G399073 | 1001 | 0.604 | 0.135 | 0.044 | 0.305 | C |
| Zm2G024668 | 351 | 0.102 | 0.06 | 0.332 | 0.501 | _ |
| Zm2G002220 | 793 | 0.963 | 0.228 | 0.007 | 0.007 | C |
| Zm5G813217 | 758 | 0.887 | 0.618 | 0.008 | 0.006 | C |

| **WoLF PSORT prediction results** | | | | | |
| --- | --- | --- | --- | --- | --- |
| Query Name | Location:Score | | |  |  |
| At5G52640 | nucl:6 | cyto: 5, | chlo: 2 |  |  |
| At5G56000 | cyto:9 | plas: 2, | pero:1 |  |  |
| At5G56010 | cyto: | plas: 2, | mito:1 |  |  |
| At5G56030 | cyto:7, | plas: 4, | nucl: 2 |  |  |
| At4G24190 | E.R.:7, | vacu: 4, | nucl: 1 | extr:1 |  |
| At2G04030 | chlo:12, | nucl: 1 |  |  |  |
| At3G07770 | mito:9.5, | chlo_mito: 6.5, | chlo:2 |  |  |
|  |  |  |  |  |  |
| Bd5g02037 | cyto:11, | mito: 1, | plas: 1 |  |  |
| Bd3g39620 | cyto:7, | nucl: 4, | chlo: 2 |  |  |
| Bd3g39590 | cyto:8, | nucl: 3, | chlo: 2 |  |  |
| Bd3g39630 | nucl:6, | cyto: 5, | chlo: 2 |  |  |
| Bd1g30130 | vacu:5, | E.R.: 5, | plas: 2 | chlo:1 |  |
| Bd4g06370 | chlo:10.5, | chlo_mito: 7.5 | mito: 3 |  |  |
| Bd4g32941 | chlo:11.5 | chlo_mito: 7, | mito:1.5 |  |  |
| Bd3g38897 | chlo:13.5, | chlo_mito: 7.5 |  |  |  |
|  |  |  |  |  |  |
| Cr09g386750 | cyto:4 | plas: 3 | vacu: 2 | E.R.:2 | chlo:1 |
| Cr12g514850 | chlo:7.5 | chlo_mito: 7.33 | mito: 6 | cyto_mito:3.83 |  |
| Cr02g080650 | chlo:5 | E.R.: 5 | mito: 2 | nucl:1 |  |
|  |  |  |  |  |  |
| Gm09G131500 | cyto:7 | nucl: 4 | plas: 2 |  |  |
| Gm16G178800 | cyto:7 | plas: 3 | nucl: 2, | mito:1 |  |
| Gm08G332900 | cyto:8 | plas: 2, | chlo: 1, | nucl:1 |  |
| Gm14G011600 | cyto:8 | nucl: 2 | plas: 2 | chlo:1 |  |
| Gm18G074100 | cyto:7 | nucl: 2 | plas: 2 | chlo:1 |  |
| Gm02G302500 | cyto:8 | nucl: 2 | plas: 2 | chlo:1 |  |
| Gm08G032900 | chlo:11.5 | chlo_mito: 7.3 | mito: 2 |  |  |
| Gm17G258700 | vacu:5 | E.R.: 5 | plas: 2 |  |  |
| Gm14G219700 | vacu:6 | E.R.: 5 | plas: 2 |  |  |
| Gm02G124500 | chlo:11.5 | chlo_mito: 7.5 | mito: 2 |  |  |
| Gm02G305600 | chlo:10.5 | chlo_mito: 7 | mito:2.5 |  |  |
| Gm01G068000 | chlo:14 |  |  |  |  |
| Gm14G007700 | chlo:7.5 | chlo_mito: 6.5 | mito:4 |  |  |
|  |  |  |  |  |  |
| Gr004G138600 | cyto:8 | nucl: 3 | chlo: 2 |  |  |
| Gr008G274600 | cyto:6 | plas: 4 | nucl: 2 | mito:1 |  |
| Gr003G155600 | cyto:10 | nucl: 3 |  |  |  |
| Gr002G103000 | cyto:8 | plas: 2 | chlo: 1 | nucl:1 |  |
| Gr004G033900 | cyto:8 | plas: 2 | chlo: 1 | nucl:1 |  |
| Gr013G150300 | cyto:8 | plas: 2 | chlo: 1 | nucl:1 |  |
| Gr004G034000 | cyto:8 | plas: 2 | chlo: 1 | nucl:1 |  |
| Gr002G122800 | vacu:4 | extr: 3 | E.R.: 3 | chlo:1, |  |
| Gr001G220600 | mito:9.5 | chlo_mito: 7 | chlo: 3 |  |  |
| Gr013G098300 | nucl:3 | extr: 3 | chlo: 2 | vacu:2 |  |
| Gr005G148100 | chlo:11.5 | chlo_mito: 6.5 | nucl: 1 |  |  |
| Gr010G003000 | plas:9 | E.R.: 3 | vacu: 2 |  |  |
|  |  |  |  |  |  |
| Mt6g452990 | cyto:8 | plas: 2, | nucl: 1 | mito:1 | E.R.:1 |
| Mt1g099840 | cyto:8 | nucl: 3 | mito: 1 | plas:1 |  |
| Mt5g096460 | cyto:8 | plas: 2 | chlo: 1, | nucl:1, | mito:1 |
| Mt5g096430 | cyto:8 | plas: 2 | chlo: 1 | nucl:1 | mito:1 |
| Mt5g097320 | chlo_mito: 6.83 | chlo:6.5, | mito: 6, | cyto_mito:3.83 |  |
| Mt1g025430 | E.R.:7 | vacu: 5 | plas: 1 |  |  |
|  |  |  |  |  |  |
| Os04g01740 | cyto:9 | chlo: 1 | mito: 1 | plas:1 | vacu:1 |
| Os08g39140 | cyto:8 | plas: 2 | chlo: 1 | nucl:1 | mito:1 |
| Os09g30412 | nucl:6 | cyto: 4 | chlo: 2 | plas:2 |  |
| Os09g30418 | nucl:7 | cyto: 4 | chlo: 1 | plas:1 |  |
| Os09g29840 | chlo:13.5 | chlo_mito: 7.5 |  |  |  |
| Os08g38086 | chlo:11.5 | chlo_mito: 7 | mito:1.5 |  |  |
| Os12g32986 | chlo:12 | mito: 1 |  |  |  |
| Os06g50300 | vacu:5 | E.R.: 5 | plas: 2 | chlo:1 |  |
|  |  |  |  |  |  |
| Pp3c156620V3 | cyto:5, | nucl: 3 | plas: 2 | vacu:2 | chlo:1 |
| Pp3c156622V3 | cyto:5 | nucl: 3 | plas: 2 | vacu:2 | chlo:1 |
| Pp3c1512510V3 | cyto:5 | nucl: 3 | plas: 2 | vacu:2 | chlo:1 |
| Pp3c1512515V3 | cyto:5 | nucl: 3 | plas: 2 | vacu:2 | chlo:1 |
| Pp3c96690V3 | nucl:10 | cyto: 3 |  |  |  |
| Pp3c96640V3 | nucl:9 | cyto: 3 | chlo: 1 |  |  |
| Pp3c154270V3 | nucl:4 | cyto: 4 | plas: 2 | vacu:2 | chlo:1 |
| Pp3c143360V3 | chlo:5 | E.R.: 4 | vacu: 3 | nucl:1 |  |
| Pp3c4810V3 | chlo:13 |  |  |  |  |
| Pp3c1222440V3 | chlo:13 |  |  |  |  |
| Pp3c1915000V3 | mito:8 | chlo: 4 | nucl: 1 |  |  |
|  |  |  |  |  |  |
| Ta2DS3B16D8173 | cyto:10 | nucl: 3 |  |  |  |
| Ta2AS67EFE0FAE | cyto:10 | nucl: 3 |  |  |  |
| Ta2BSF828BA5F41 | cyto:9 | chlo: 3 | nucl: 1 |  |  |
| Ta2BSF828BA5F4 | cyto:9 | chlo: 3 | nucl: 1 |  |  |
| Ta7BS1A6D16C6B | cyto:7 | nucl: 4, | chlo: 2 |  |  |
| Ta7DSCB359539B | cyto:7 | nucl: 4, | chlo: 2 |  |  |
| Ta5DL89CF7F5DE | cyto:6, | nucl: 5, | chlo: 2 |  |  |
| Ta5BL0F3A986F9 | cyto:6, | nucl: 5 | chlo: 2 |  |  |
| Ta7AS76670DCAB | cyto:11 | nucl: 2 |  |  |  |
| Ta5AL0C2D144B0 | cyto:11 | nucl: 2 |  |  |  |
| Ta5BL37ECD3B1E | cyto:8 | nucl: 4 | pero: 1 |  |  |
| Ta5DL5A546D5B3 | cyto:8 | nucl: 4 | pero: 1 |  |  |
| Ta5AS5B7BFBD23 | cyto:7 | nucl: 3 | chlo: 1 | vacu:1 | pero:1 |
| Ta7BL870E640C5 | cyto:7 | nucl: 4 | extr: 1 | cysk:1 |  |
| Ta5AL1DA3B4631 | cyto:7 | nucl: 5 | pero: 1 |  |  |
| Ta5DSAC5D29D23 | chlo:9.5 | chlo_mito: 7.5 | mito:4 |  |  |
| Ta7AL1A21E8798 | cyto:5 | nucl: 2 | golg: 2 | chlo:1 | plas:1 |
| Ta5BSAB86BB5DE | chlo:9.5 | chlo_mito: 7.5 | mito:4 |  |  |
|  |  |  |  |  |  |
| Zm5G833699 | cyto:9 | chlo: 1 | nucl: 1 | plas:1 | vacu:1 |
| Zm2G069651 | nucl:6 | cyto: 5 | chlo: 2 |  |  |
| Zm2G112165 | cyto:6 | nucl: 5 | chlo: 2 |  |  |
| Zm2G012631 | nucl:7 | cyto: 4 | chlo: 2 |  |  |
| Zm2G141931 | E.R.:5 | chlo: 3, | vacu: 3 | plas:2 |  |
| Zm2G399073 | nucl:7 | cyto: 4 | chlo: 1 | mito:1 |  |
| Zm2G024668 | cyto:8 | nucl: 4 | cysk: 1 |  |  |
| Zm2G002220 | chlo:13.5 | chlo_mito: 7.5 |  |  |  |
| Zm5G813217 | chlo:13.5 | chlo_mito: 7.5 |  |  |  |

| **Predotar v. 1.03 prediction results** | | | | | |
| --- | --- | --- | --- | --- | --- |
| Sequence | Mitochondrial | Plastid | ER | Elsewher | Prediction |
| At5G52640 | 0,01 | 0,01 | 0,00 | 0,98 | none |
| At5G56000 | 0,01 | 0,01 | 0,00 | 0,99 | none |
| At5G56010 | 0,01 | 0,01 | 0,00 | 0,99 | none |
| At5G56030 | 0,01 | 0,01 | 0,00 | 0,98 | none |
| At4G24190 | 0,02 | 0,00 | 0,99 | 0,01 | ER |
| At2G04030 | 0,39 | 0,83 | 0,03 | 0,10 | plastid |
| At3G07770 | 0,92 | 0,00 | 0,00 | 0,08 | mitochondrial |
|  |  |  |  |  |  |
| Bd5g02037 | 0,01 | 0,00 | 0,00 | 0,99 | none |
| Bd3g39620 | 0,01 | 0,01 | 0,01 | 0,97 | none |
| Bd3g39590 | 0,01 | 0,01 | 0,01 | 0,97 | none |
| Bd3g39630 | 0,01 | 0,01 | 0,01 | 0,97 | none |
| Bd1g30130 | 0,02 | 0,00 | 0,99 | 0,01 | ER |
| Bd4g06370 | 0,78 | 0,01 | 0,01 | 0,22 | mitochondrial |
| Bd4g32941 | 0,35 | 0,45 | 0,02 | 0,35 | plastid |
| Bd3g38897 | 0,14 | 0,85 | 0,00 | 0,13 | plastid |
|  |  |  |  |  |  |
| Cr09g386750 | 0,01 | 0,02 | 0,00 | 0,98 | none |
| Cr12g514850 | 0,13 | 0,87 | 0,01 | 0,11 | plastid |
| Cr02g080650 | 0,01 | 0,00 | 0,99 | 0,01 | ER |
|  |  |  |  |  |  |
| Gm09G131500 | 0,01 | 0,01 | 0,00 | 0,99 | none |
| Gm16G178800 | 0,01 | 0,01 | 0,00 | 0,99 | none |
| Gm08G332900 | 0,01 | 0,00 | 0,00 | 0,99 | none |
| Gm14G011600 | 0,01 | 0,01 | 0,02 | 0,97 | none |
| Gm18G074100 | 0,01 | 0,00 | 0,00 | 0,99 | none |
| Gm02G302500 | 0,01 | 0,01 | 0,02 | 0,97 | none |
| Gm08G032900 | 0,54 | 0,00 | 0,11 | 0,41 | mitochondrial |
| Gm17G258700 | 0,03 | 0,00 | 0,99 | 0,01 | ER |
| Gm14G219700 | 0,03 | 0,00 | 0,99 | 0,01 | ER |
| Gm02G124500 | 0,46 | 0,84 | 0,03 | 0,08 | plastid |
| Gm02G305600 | 0,60 | 0,07 | 0,08 | 0,34 | mitochondrial |
| Gm01G068000 | 0,36 | 0,86 | 0,03 | 0,09 | plastid |
| Gm14G007700 | 0,52 | 0,27 | 0,04 | 0,34 | mitochondrial |
|  |  |  |  |  |  |
| Gr004G138600 | 0,01 | 0,03 | 0,00 | 0,96 | none |
| Gr008G274600 | 0,01 | 0,03 | 0,00 | 0,97 | none |
| Gr003G155600 | 0,01 | 0,02 | 0,00 | 0,98 | none |
| Gr002G103000 | 0,01 | 0,01 | 0,00 | 0,99 | none |
| Gr004G033900 | 0,01 | 0,00 | 0,00 | 0,99 | none |
| Gr013G150300 | 0,01 | 0,00 | 0,00 | 0,99 | none |
| Gr004G034000 | 0,01 | 0,00 | 0,00 | 0,99 | none |
| Gr002G122800 | 0,01 | 0,00 | 0,99 | 0,01 | ER |
| Gr001G220600 | 0,91 | 0,00 | 0,00 | 0,09 | mitochondrial |
| Gr013G098300 | 0,01 | 0,00 | 0,93 | 0,07 | ER |
| Gr005G148100 | 0,44 | 0,65 | 0,06 | 0,19 | plastid |
| Gr010G003000 | 0,02 | 0,00 | 0,99 | 0,01 | ER |
|  |  |  |  |  |  |
| Mt6g452990 | 0,01 | 0,01 | 0,00 | 0,99 | none |
| Mt1g099840 | 0,01 | 0,01 | 0,00 | 0,99 | none |
| Mt5g096460 | 0,01 | 0,00 | 0,00 | 0,99 | none |
| Mt5g096430 | 0,01 | 0,00 | 0,00 | 0,99 | none |
| Mt5g097320 | 0,44 | 0,00 | 0,08 | 0,52 | mitochondrial |
| Mt1g025430 | 0,03 | 0,00 | 0,99 | 0,01 | ER |
|  |  |  |  |  |  |
| Os04g01740 | 0,01 | 0,01 | 0,00 | 0,98 | none |
| Os08g39140 | 0,01 | 0,01 | 0,01 | 0,97 | none |
| Os09g30412 | 0,01 | 0,01 | 0,01 | 0,97 | none |
| Os09g30418 | 0,01 | 0,01 | 0,01 | 0,97 | none |
| Os09g29840 | 0,64 | 0,56 | 0,01 | 0,16 | mitochondrial |
| Os08g38086 | 0,50 | 0,20 | 0,01 | 0,40 | mitochondrial |
| Os12g32986 | 0,87 | 0,04 | 0,00 | 0,13 | mitochondrial |
| Os06g50300 | 0,04 | 0,00 | 0,99 | 0,01 | ER |
|  |  |  |  |  |  |
| Pp3c156620V3 | 0,01 | 0,02 | 0,00 | 0,98 | none |
| Pp3c156622V3 | 0,01 | 0,02 | 0,00 | 0,98 | none |
| Pp3c1512510V3 | 0,01 | 0,01 | 0,00 | 0,98 | none |
| Pp3c1512515V3 | 0,01 | 0,01 | 0,00 | 0,98 | none |
| Pp3c96690V3 | 0,01 | 0,01 | 0,00 | 0,98 | none |
| Pp3c96640V3 | 0,01 | 0,01 | 0,00 | 0,98 | none |
| Pp3c154270V3 | 0,01 | 0,01 | 0,00 | 0,98 | none |
| Pp3c143360V3 | 0,09 | 0,00 | 0,95 | 0,04 | ER |
| Pp3c4810V3 | 0,31 | 0,91 | 0,01 | 0,06 | plastid |
| Pp3c1222440V3 | 0,01 | 0,97 | 0,07 | 0,03 | plastid |
| Pp3c1915000V3 | 0,82 | 0,00 | 0,02 | 0,17 | mitochondrial |
|  |  |  |  |  |  |
| Ta2DS3B16D8173 | 0,01 | 0,01 | 0,00 | 0,98 | none |
| Ta2AS67EFE0FAE | 0,09 | 0,00 | 0,00 | 0,91 | none |
| Ta2BSF828BA5F41 | 0,01 | 0,00 | 0,00 | 0,99 | none |
| Ta2BSF828BA5F4 | 0,01 | 0,00 | 0,00 | 0,99 | none |
| Ta7BS1A6D16C6B | 0,01 | 0,01 | 0,02 | 0,97 | none |
| Ta7DSCB359539B | 0,01 | 0,01 | 0,02 | 0,97 | none |
| Ta5DL89CF7F5DE | 0,01 | 0,01 | 0,01 | 0,97 | none |
| Ta5BL0F3A986F9 | 0,01 | 0,01 | 0,01 | 0,97 | none |
| Ta7AS76670DCAB | 0,01 | 0,00 | 0,00 | 0,99 | none |
| Ta5AL0C2D144B0 | 0,01 | 0,00 | 0,00 | 0,99 | none |
| Ta5BL37ECD3B1E | 0,01 | 0,03 | 0,00 | 0,97 | none |
| Ta5DL5A546D5B3 | 0,01 | 0,03 | 0,00 | 0,97 | none |
| Ta5AS5B7BFBD23 | 0,01 | 0,01 | 0,00 | 0,99 | none |
| Ta7BL870E640C5 | 0,01 | 0,01 | 0,00 | 0,99 | none |
| Ta5AL1DA3B4631 | 0,01 | 0,03 | 0,00 | 0,97 | none |
| Ta5DSAC5D29D23 | 0,75 | 0,01 | 0,01 | 0,24 | mitochondrial |
| Ta7AL1A21E8798 | 0,01 | 0,01 | 0,00 | 0,99 | none |
| Ta5BSAB86BB5DE | 0,76 | 0,01 | 0,02 | 0,23 | mitochondrial |
|  |  |  |  |  |  |
| Zm5G833699 | 0,01 | 0,00 | 0,00 | 0,99 | none |
| Zm2G069651 | 0,01 | 0,01 | 0,01 | 0,97 | none |
| Zm2G112165 | 0,01 | 0,01 | 0,01 | 0,97 | none |
| Zm2G012631 | 0,01 | 0,01 | 0,01 | 0,97 | none |
| Zm2G141931 | 0,03 | 0,00 | 0,99 | 0,01 | ER |
| Zm2G399073 | 0,01 | 0,01 | 0,00 | 0,99 | none |
| Zm2G024668 | 0,01 | 0,01 | 0,01 | 0,97 | none |
| Zm2G002220 | 0,06 | 0,97 | 0,01 | 0,03 | plastid |
| Zm5G813217 | 0,48 | 0,60 | 0,02 | 0,21 | plastid |
